# Prognostic impact of c-Rel nuclear expression and *REL* amplification and crosstalk between c-Rel and the p53 pathway in diffuse large B-cell lymphoma

**DOI:** 10.18632/oncotarget.4319

**Published:** 2015-06-30

**Authors:** Ling Li, Zijun Y. Xu-Monette, Chi Young Ok, Alexandar Tzankov, Ganiraju C. Manyam, Ruifan Sun, Carlo Visco, Mingzhi Zhang, Santiago Montes-Moreno, Karen Dybkaer, April Chiu, Attilio Orazi, Youli Zu, Govind Bhagat, Kristy L. Richards, Eric D. Hsi, William W.L. Choi, J. Han van Krieken, Jooryung Huh, Maurilio Ponzoni, Andrés J.M. Ferreri, Michael B. Møller, Jinfeng Wang, Ben M. Parsons, Jane N. Winter, Miguel A. Piris, Lan V. Pham, L. Jeffrey Medeiros, Ken H. Young

**Affiliations:** ^1^ Zhengzhou University, The First Affiliated University Hospital, Zhengzhou, China; ^2^ Department of Hematopathology, The University of Texas MD Anderson Cancer Center, Houston, TX, USA; ^3^ University Hospital, Basel, Switzerland; ^4^ Department of Bioinformatics and Computational Biology, The University of Texas MD Anderson Cancer Center, Houston, TX, USA; ^5^ San Bortolo Hospital, Vicenza, Italy; ^6^ Hospital Universitario Marques de Valdecilla, Santander, Spain; ^7^ Aalborg Univwersity Hospital, Aalborg, Denmark; ^8^ Memorial Sloan-Kettering Cancer Center, New York, NY, USA; ^9^ Weill Medical College of Cornell University, New York, NY, USA; ^10^ The Methodist Hospital, Houston, TX, USA; ^11^ Columbia University Medical Center and New York Presbyterian Hospital, New York, NY, USA; ^12^ University of North Carolina School of Medicine, Chapel Hill, NC, USA; ^13^ Cleveland Clinic, Cleveland, OH, USA; ^14^ University of Hong Kong Li Ka Shing Faculty of Medicine, Hong Kong, China; ^15^ Radboud University Nijmegen Medical Centre, Nijmegen, Netherlands; ^16^ Asan Medical Center, Ulsan University College of Medicine, Seoul, Korea; ^17^ San Raffaele H. Scientific Institute, Milan, Italy; ^18^ Odense University Hospital, Odense, Denmark; ^19^ Shanxi Cancer Hospital, Shanxi, China; ^20^ Gundersen Medical Foundation, La Crosse, WI, USA; ^21^ Feinberg School of Medicine, Northwestern University, Chicago, IL, USA; ^22^ The University of Texas School of Medicine, Graduate School of Biomedical Sciences, Houston, Texas, USA

**Keywords:** c-Rel, NF-kB, p53, DLBCL, gene expression profiling

## Abstract

Dysregulated NF-κB signaling is critical for lymphomagenesis. The regulation, function, and clinical relevance of c-Rel/NF-κB activation in diffuse large B-cell lymphoma (DLBCL) have not been well studied. In this study we analyzed the prognostic significance and gene-expression signature of c-Rel nuclear expression as surrogate of c-Rel activation in 460 patients with *de novo* DLBCL. Nuclear c-Rel expression, observed in 137 (26.3%) DLBCL patients frequently associated with extranoal origin, did not show significantly prognostic impact in the overall- or germinal center B-like-DLBCL cohort, likely due to decreased pAKT and Myc levels, up-regulation of *FOXP3, FOXO3, MEG3* and other tumor suppressors coincided with c-Rel nuclear expression, as well as the complicated relationships between NF-κB members and their overlapping function. However, c-Rel nuclear expression correlated with significantly poorer survival in p63^+^ and BCL-2^−^ activated B-cell-like-DLBCL, and in DLBCL patients with *TP53* mutations. Multivariate analysis indicated that after adjusting clinical parameters, c-Rel positivity was a significantly adverse prognostic factor in DLBCL patients with wild type *TP53*. Gene expression profiling suggested dysregulations of cell cycle, metabolism, adhesion, and migration associated with c-Rel activation. In contrast, *REL* amplification did not correlate with c-Rel nuclear expression and patient survival, likely due to co-amplification of genes that negatively regulate NF-κB activation. These insights into the expression, prognostic impact, regulation and function of c-Rel as well as its crosstalk with the p53 pathway underscore the importance of c-Rel and have significant therapeutic implications.

## INTRODUCTION

Diffuse large B-cell lymphoma (DLBCL) is a heterogeneous aggressive non-Hodgkin lymphoma that can be classified into germinal center B-like (GCB) or activated B-cell-like (ABC) DLBCL. Aberrant activation of nuclear factor-kappaB (NF-κB), either through the “canonical” pathway activating p50/p65 and p50/c-Rel dimers, or through the “non-canonical” pathway activating p52/RelB dimers, has been associated with tumor proliferation and survival in DLBCL, especially in the ABC subtype [1, 2]. The canonical and non-canonical pathways are generally believed to be independent; however, the non-canonical pathway may attenuate activities of the canonical pathways [3].

c-Rel encoded by the *REL* gene is a unique NF-κB member, predominantly expressed in lymphoid and myeloid tissues, likely contributed by the unique regulators for c-Rel activation. NF-κB inhibitor IκBα preferentially inhibits p65/p50 dimers, whereas IκBε controls p65/c-Rel, and c-Rel activation also depends on the non-redundant regulator IκBβ [4–7], and the protease activities of MALT1 [8]. MALT1 inhibitors specifically impair c-Rel nuclear localization and display selective activity against ABC-DLBCL *ex vivo* [9]. In addition, novel IκB kinase (IKK)-dependent and proteasomal-independent pathway was found to degrade IκBα and activate p50/c-Rel in B-cells [10], triggered by stimuli different from the non-canonical NF-κB pathway. However, little is known about whether and how the upstream stimuli for NF-κB activation, e.g., signaling through B-cell receptors (BCR), T-cell receptors (TCR), tumor-necrosis factor (TNF) receptors, Toll-like receptors (TLR), and mitogen-activated protein kinases (MAPK) [11], differentially regulate c-Rel and other NF-κB subunits.

c-Rel has both nonredundant and overlapping functions compared with p65 and p50. c-Rel regulates cytokine production and plays an important role in proliferation and inflammation mainly regulating development of T-cells [12–14]. c-Rel promotes cell survival by transactivating antiapoptotic and cell cycle genes, such as *BCLXL*/*BCL2L1, BCL2A1, XIAP, cIAP*, and *cyclins* [4, 15, 16]. In addition, during GC reaction in B-cell development, c-Rel is required for B-cell activation before GC formation and maintenance of the GC reaction by regulating metabolism, fueling proliferation independent of Myc [17], and is crucial for the development of follicular helper T cells [7, 18]. c-Rel-knockout mice are viable but have deficiencies in immune responses [4, 19]. However, c-Rel, but not other NF-κB members, has a unique ability to transform avian lymphoid cells *in vitro* [4], and is associated with increased lymphoma risk *in vivo* [20].

c-Rel functions are also affected by the p53 pathway. In mouse models the requirement for NF-κB signaling in tumor development depends on the p53 status [21]. Wild-type (WT-) p53 and NF-κB antagonize each other, however NF-κB can also enhance p53 stability and activities in some circumstances [22]. In contrast, p53 mutants (MUT-p53) cooperate with NF-κB to promote tumor invasion and metastasis [23, 24]. p53 can also directly regulate NF-κB expression and activation. WT-p53 negatively regulates NF-κB activation and function [25, 26], whereas MUT-p53 induces *p52*/*NFKB2* gene expression [27]. Moreover, crosstalk also exists between NF-κB and p63, another member of the p53 family [28–30]. Overexpression of ΔNp63α leads to increased c-Rel expression, and epidermal hyperplasia and diffuse inflammation in transgenic mice [28]. The ΔNp63α–c-Rel complex represses *CDKN1A/p21* and promotes epithelial cell proliferation in human squamous carcinoma cells [29]. In head and neck squamous cell carcinoma with MUT-p53, c-Rel overexpression activated by TNF-α modulates ΔNp63α/Tap73 interactions and their function, promoting proliferation and cell survival [30].

c-Rel has been proposed to be an attractive therapeutic target, whose inhibition can suppress tumor growth without causing systemic tissue toxicity [19]. A study group showed that c-Rel inhibition is a novel strategy to ameliorate GVHD reduced alloactivation without compromising T-cell mediated immune responses [31], and a small molecule c-Rel inhibitor had anti-proliferative effect in both GCB- and ABC-DLBCL cell lines [32]. Since *REL* gene was found frequently amplified in DLBCL (~15%), *REL* activation may play a role in lymphomagenesis, which however, was not supported by immunofluorescence analysis [33]. One study of 68 *de novo* DLBCL cases found that 15 GCB-DLBCL cases positive for c-Rel nuclear expression by immunohistochemistry had worse survival compared to 9 GCB-DLBCL cases negative for c-Rel nuclear expression (*P* = 0.045) [34]. In contrast, another study using *a* > 30% cutoff for c-Rel nuclear staining showed that 57 c-Rel^+^ DLBCL patients had significantly better overall survival than 31 c-Rel^−^ DLBCL patients [35]. Large scale studies of *REL* amplification and c-Rel nuclear expression, and the prognostic impact of concurrent dysregulation of NF-κB and *TP53* [36] in DLBCL are lacking. In this study, we aimed to evaluate the clinical significance of c-Rel nuclear expression and *REL* amplification in DLBCL patients, to gain insight into the underlying biology, c-Rel function, activation mechanisms, and relationship with other NF-κB subunits.

## RESULTS

### c-Rel nuclear expression and correlation with nuclear expression of other NF-κB subunits

**Figure 1:**
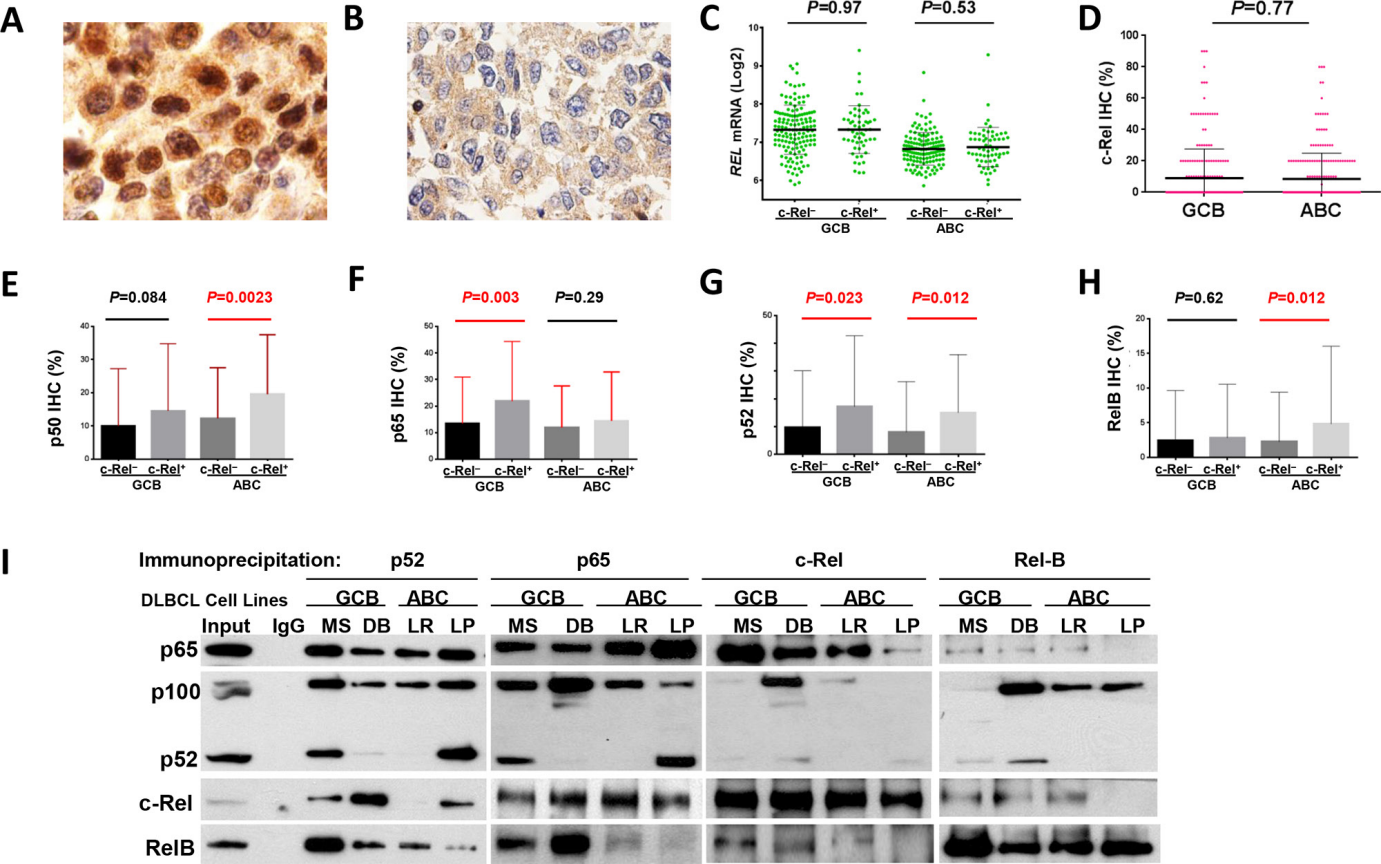
Nuclear expression of c-Rel and other NF-κB subunits **A–B.** Representative immunohistochemical staining for positive and negative nuclear c-Rel expression. **C.** Nuclear c-Rel positivity did not correlate with *REL* mRNA levels in GCB- and ABC-DLBCL. **D.** Expression levels of nuclear c-Rel did not show significant difference between GCB- and ABC-DLBCL. **E-H.** Association of c-Rel positivity with nuclear expression of other NF-κB subunits. Note: red lines indicate upregulation whereas blue lines indicated downregulation with significant or border-line *P* values. **I.** Dimerization of NF-κB subunits in DLBCL *in vitro*. Nuclear extract purified from MS and DB (GCB), and LR and LP (ABC) cells were subjected to coimmunoprecipitation analysis with p65, c-Rel, p52, and RelB antibodies. Normal rabbit IgG was used as a nonspecific negative control. Precipitated immune-complexes were subjected to Western blot analysis with p65, c-Rel, p52, and RelB antibodies.

Immunohistochemistry was used to analyze the nuclear expression of c-Rel, as the surrogate marker for c-Rel activation [34] (Fig. 1A). A cutoff of ≥ 5% of tumor cells with positive c-Rel staining nuclei was used to identify positive c-Rel nuclear expression (c-Rel^+^). Using this cutoff, 137 patients (26.3%) of the 460 successfully stained cases had c-Rel^+^ DLBCL, with different expression levels (5–90% of the tumor cells with c-Rel^+^ nuclei), whereas majority (73.7%) of the cases were negative for c-Rel nuclear expression with or without cytoplasmic staining (Fig. 1B, Supplementary Fig. S1A). The mean expression level of nuclear c-Rel was significantly lower than those of nuclear p65 and p50 in our cohort (Supplementary Fig. S1B).

Nuclear c-Rel positivity did not correlate with *REL* mRNA levels by Spearman rank correlation (*r* = 0.004, *P* = 0.94), either in GCB- or ABC-DLBCL (Fig. 1C). Consistent with a previous study [35], GCB- and ABC-DLBCL had similar level of nuclear c-Rel protein expression (Fig. 1D). However, *REL* mRNA was significantly higher in the GCB subtype (*P* < 0.0001), even after exclusion of cases with *REL* amplification or potential gains due to polysomies (Supplementary Fig. S1C).

Nuclear expression levels of c-Rel correlated positively with expression of other NF-κB subunits (significant for p52 and RelB by Spearman rank correlation): p50 (*r* = 0.12, *P* = 0.12), p52 (*r* = 0.26, *P* = 0.724E-8), p65 (*r* = 0.085, *P* = 0.073), and RelB (*r* = 0.12, *P* = 0.013). c-Rel^+^ correlated with significantly higher levels of nuclear p65 and p52 in GCB-DLBCL, and p50, p52 and RelB in ABC-DLBCL (Fig. 1E-H). At the mRNA level, c-Rel^+^ correlated with upregulation of *NFKB1* and *RELA* (but not *NFKB2* or *RELB*) in ABC-DLBCL (Supplementary Fig. S1D-E). It has been known that c-Rel predominately forms dimers with p50 [1]. Our coimmunoprecipitation analysis using nuclear extracts purified from representative human derived GCB- and ABC-DLBCL cell lines (MS, DB, LR, LP) however showed that c-Rel predominantly binds to p65 in all cell lines (more intense in GCB than ABC cell lines); and that in some cell lines, p52 and RelB also formed dimers with c-Rel but to a lesser extent (Fig. 1I).

### Prognostic impact of c-Rel nuclear expression

#### Clinicopathologic features

The clinicopathologic features of the study cohort are shown in Table 1. Interestingly, the c-Rel^+^ DLBCL group had a higher proportion of patients with extranodal disease (*P* = 0.0033), and had no association with other clinical parameters. Pathologically, the c-Rel^+^ compared to the c-Rel^−^ DLBCL group less frequently had Myc or pAKT overexpression, whereas more frequently expressed nuclear p50, p52 and RelB (Table 1).

**Table 1:** Clinicopathologic characteristics of 460 *de novo* DLBCL patients treated with R-CHOP

Variables	DLBCL		*P*	GCB-DLBCL		*P*	ABC-DLBCL		*P*
	c-Rel^+^	c-Rel^−^		c-Rel^+^	c-Rel^−^		c-Rel^+^	c-Rel^−^	
	*N* (%)	*N* (%)		*N* (%)	*N* (%)		*N* (%)	*N* (%)	
**Patients**	137 (100)	323 (100)		66 (100)	165 (100)		70 (100)	158 (100)	.68
**Gender**									
Male	78 (57)	192 (59)	.61	31 (47)	104 (63)	**.025**	46 (66)	88 (56)	.16
Female	59 (43)	131 (41)		35 (53)	61 (37)		24 (34)	70 (44)	
**Age (yr)**									
<60	52 (38)	140 (43)	.30	29 (44)	86 (52)	.31	22 (31)	54 (34)	.76
≥60	85 (62)	183 (57)		37 (56)	79 (48)		48 (69)	104 (66)	
**Stage**									
I-II	65 (50)	143 (46)	.39	36 (57)	84 (53)	.56	28 (42)	59 (38)	.54
III-IV	65 (50)	171 (54)		27 (43)	75 (47)		38 (58)	96 (62)	
**B-symptoms**									
No	88 (71)	196 (63)	.099	46 (79)	108 (68)	.09	42 (65)	88 (58)	.33
Yes	36 (29)	117 (37)		12 (21)	52 (32)		23 (35)	65 (42)	
**LDH**									
Normal	46 (40)	113 (37)	.56	24 (44)	57 (37)	.37	22 (37)	56 (37)	.98
Elevated	69 (60)	193 (63)		31 (56)	98 (63)		37 (63)	95 (63)	
**# of extranodal sites**									
0-1	103 (80)	235 (75)	.31	49 (80)	121 (77)	.55	54 (81)	114 (74)	.29
≥2	26 (20)	77 (25)		12 (20)	37 (23)		13 (19)	40 (26)	
**Performance status**									
0-1	95 (86)	247 (83)	.47	48 (94)	125 (83)	.055	46 (78)	122 (82)	.52
≥2	16 (14)	52 (17)		3 (6)	25 (17)		13 (22)	27 (18)	
**Size of largest tumor**									
<5 cm	47 (53)	149 (58)	.38	22 (51)	78 (61)	.24	25 (54)	71 (55)	.94
≥5cm	42 (47)	107 (42)		21 (49)	49 (39)		21 (46)	58 (45)	
**IPI risk group**									
0-2	83 (65)	189 (60)	.32	46 (75)	104 (65)	.14	36 (54)	85 (54)	.99
3-5	45 (35)	127 (40)		15 (25)	56 (35)		30 (46)	71 (46)	
**Therapy response**									
CR	103 (75)	247 (77)	.77	51 (77)	121 (73)	.54	51 (73)	126 (80)	.25
PR	17	42		7	20		10	22	
SD	6	13		4	8		2	5	
PD	11	21		4	16		7	5	
**Primary origin**									
Nodal	70 (53)	222 (69)	**.0015**	36 (57)	113 (69)	.095	34 (51)	109 (69)	**.0077**
Extranodal	61 (47)	99 (31)		27 (43)	51 (31)		33 (49)	48 (31)	
**Ki-67**									
<70%	49 (36)	113 (35)	1.0	27 (41)	64 (39)	.88	22 (31)	49 (31)	1.0
≥70%	88 (64)	207 (65)		39 (59)	98 (61)		48 (69)	109 (69)	
***TP53* mutation**									
WT *TP53*	91 (74)	222 (78)	.45	42 (71)	107 (72)	1.0	49 (77)	115 (84)	.24
MUT *TP53*	32 (26)	64 (22)		17 (29)	42 (28)		15 (23)	22 (16)	
***MYC* translocation**									
−	93 (91)	180 (86)	.27	44 (94)	77 (78)	**.0019**	49 (89)	103 (94)	.36
+	9 (9)	29 (14)		3 (6)	22 (22)		6 (11)	7 (6)	
***BCL2* translocation**									
−	103 (81)	208 (82)	1.0	43 (69)	82 (66)	.63	60 (92)	126 (97)	.16
+	24 (19)	47 (18)		19 (31)	43 (34)		5 (8)	4 (3)	
***BCL6* translocation**									
−	75 (70)	144 (65)	.37	43 (83)	82 (72)	.14	31 (57)	62 (58)	.95
+	32 (30)	77 (35)		9 (17)	32 (28)		23 (43)	45 (42)	
**Nuclear p50**									
−	50 (37)	168 (53)	**.0014**	32 (49)	102 (62)	.076	18 (26)	66 (44)	**.016**
+	86 (63)	147 (47)		34 (51)	62 (38)		51 (74)	85 (56)	
**Nuclear p52**									
−	77 (57)	231 (77)	**<.0001**	38 (59)	115 (76)	**.022**	39 (56)	116 (79)	**.0007**
+	58 (43)	68 (23)		26 (41)	37 (24)		31 (44)	31 (21)	
**Nuclear p65**									
−	48 (35)	135 (43)	.12	21 (32)	65 (41)	.23	27 (39)	70 (45)	.39
+	89 (65)	179 (57)		45 (68)	93 (59)		43 (61)	86 (55)	
**Nuclear RelB**									
−	109 (81)	266 (88)	.056	54 (83)	139 (89)	.28	54 (78)	127 (87)	.11
+	26 (19)	37 (12)		11 (17)	18 (11)		15 (22)	19 (13)	

**Abbreviations**: DLBCL, diffuse large B-cell lymphoma; GCB, germinal center B-cell like; ABC, activated B-cell like; LDH, lactate dehydrogenase; IPI, international prognostic index; CR, complete remission; PR, partial response; SD, stable disease; PD, progressive disease.

Moreover, dividing into GCB and ABC subtypes, c-Rel^+^ GCB-DLBCL was associated with female sex, low ECOG performance status score, and less *MYC* translocations compared with c-Rel^−^ GCB-DLBCL, whereas in the ABC subtype, c-Rel^+^ ABC-DLBCL was associated with extranodal disease (Table 1).

#### Univariate survival analysis in various DLBCL molecular subsets

**Figure 2:**
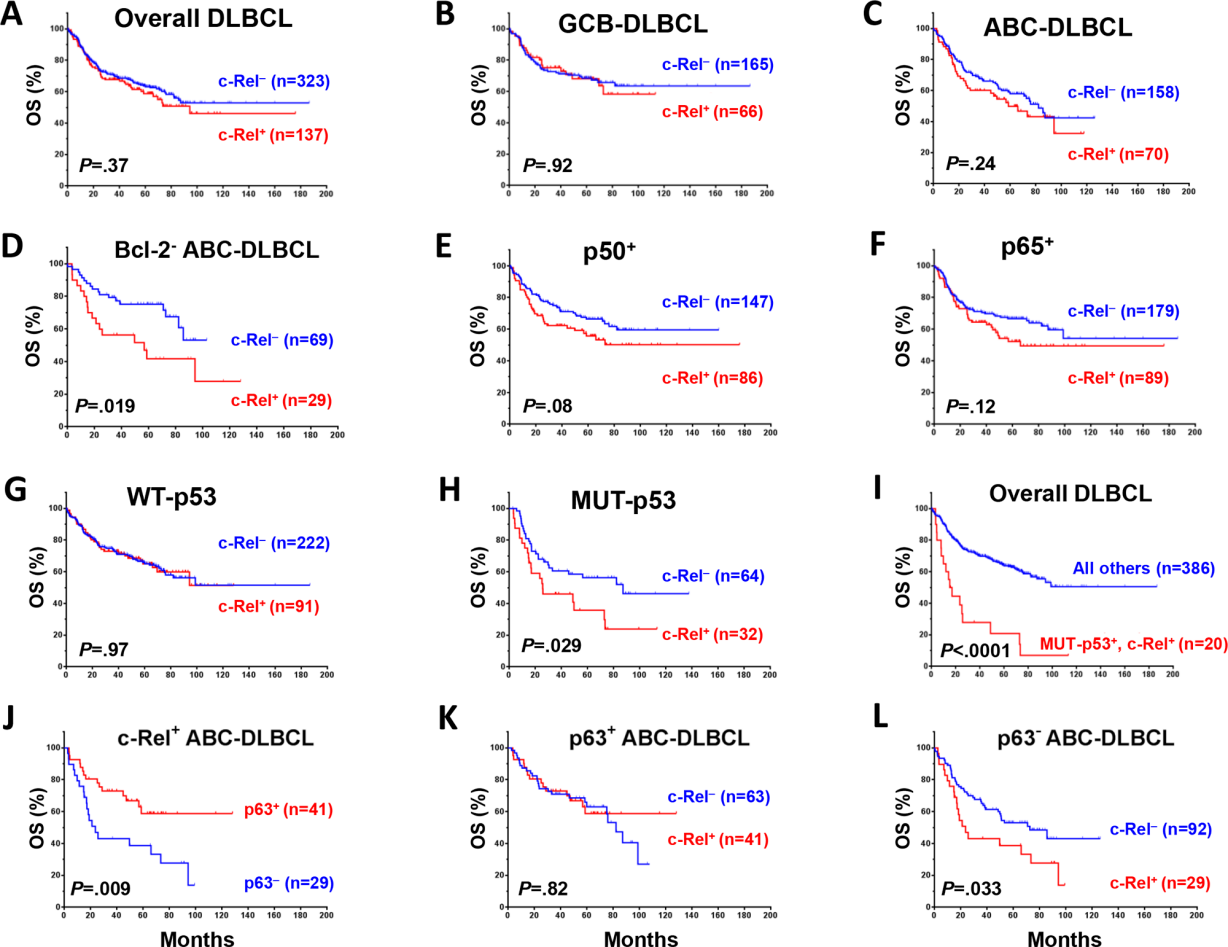
Prognostic significance of c-Rel nuclear expression in various DLBCL subsets **A–B.** In the overall- and GCB-DLBCL, c-Rel positivity did not correlate with patient survival. **C.** In ABC-DLBCL, c-Rel^+^ as a univariate did not correlate significantly with patient survival although a trend toward poorer survival was suggested. **D.** In Bcl-2^−^ (<70%) ABC-DLBCL, c-Rel^+^ correlated with significantly poorer patient survival. **E–F.** c-Rel^+^ concurrent with p50 or p65 expression correlated with poorer patient survival with marginal *P* values. **G–I.** In DLBCL with MUT-p53 but not WT-p53, c-Rel^+^ correlated with significantly poorer patient survival. **J.** In ABC-DLBCL with c-Rel nuclear expression, p63 expression correlated with significantly better patient survival. **K–L.** Only in p63^−^ but not p63^+^ ABC-DLBCL subcohort, c-Rel positivity correlated with significantly poorer patient survival. Abbreviations: OS, overall survival; PFS, Progression-free survival.

c-Rel nuclear expression did not correlate with patient survival in the overall- or GCB-DLBCL, whereas c-Rel^+^ ABC-DLBCL tended to have a poorer survival (Fig. 2A-2C). However, in DLBCL especially in ABC-DLBCL with low Bcl-2 (<70%), c-Rel^+^ correlated significantly with poorer survival (Fig. 2D). In an effort to identify the functionally relevant c-Rel dimers, we examined the prognostic impact of c-Rel^+^ within the following DLBCL subsets: p50^−^, p65^−^, p52^−^, RelB^−^, p50^+^, p65^+^, p52^+^ and RelB^+^. c-Rel^+^ DLBCL showed trends toward poorer survival only within the p50^+^ and p65^+^ subsets but not in other subsets (Fig. 2E-2F).

c-Rel nuclear expression did not correlate with survival in DLBCL patients with WT-p53 (however, the c-Rel^+^ compared with the c-Rel^−^ group had a small proportion of patients with stage III/IV disease, Table 2). In contrast, among DLBCL patients with MUT-p53, c-Rel^+^ correlated with significantly worse survival (Fig. 2G-2H). The prognostic impact of c-Rel positivity in the p65^−^, p65^+^, p50^−^ and p50^+^ subsets with WT-p53 or MUT-p53 was shown in Supplementary Figure S2A-2H. Among all the DLBCL patients, c-Rel nuclear expression concurrent with *TP53* mutations significantly predicted poorer survival (Fig. 2I). Moreover, p63 appears to be another tumor suppressor beside WT-p53 in suppressing the adverse impact of c-Rel activation, suggested by the correlation of p63 expression with better survival in c-Rel^+^ ABC-DLBCL (Fig. 2J), and a similar trend in GCB-DLBCL patients (*P* = 0.18). However, the favorable correlation of p63 expression in c-Rel^+^ ABC-DLBCL was abrogated by *TP53* mutations (Supplementary Fig. S2K). Conversely, c-Rel conferred significantly poorer survival in p63^−^ but not in p63^+^ ABC-DLBCL (Fig. 2K-L). In patients with p63^+^ ABC-DLBCL, c-Rel conferred significantly poorer survival when concurrent with *TP53* mutations (Supplementary Fig. S2K).

**Table 2:** Clinicopathologic characteristics of c-Rel^+^ *versus* c-Rel^−^ DLBCL patients with wild type (WT) or mutated (MUT) p53

Variables	WT-p53 c-Rel^+^	WT-p53 c-Rel^−^	*P*	MUT-p53 c-Rel^+^	MUT-p53 c-Rel^−^	*P*
	N (%)	N (%)		N (%)	N (%)	
**Patients**	91 (100)	222 (100)		32 (100)	64 (100)	.45
**Gender**						
Male	52 (57)	135 (61)	.55	21 (66)	35 (55)	.31
Female	39 (43)	87 (39)		11 (34)	29 (45)	
**Age (yr)**						
<60	34 (38)	89 (40)	.70	11 (34)	27 (42)	.51
≥60	57 (62)	133 (60)		21 (26)	37 (58)	
**Stage**						
I-II	46 (55)	92 (43)	.067	16 (50)	29 (45)	.66
III-IV	38 (45)	122 (57)		16 (50)	35 (55)	
**B-symptoms**						
No	61 (74)	138 (65)	.10	21 (75)	40 (63)	.24
Yes	21 (26)	76 (35)		7 (25)	24 (37)	
**Serum LDH**						
Normal	35 (49)	82 (39)	.16	9 (31)	22 (36)	.64
Elevated	37 (51)	127 (61)		20 (69)	39 (64)	
**# of extranodal sites**						
0–1	70 (83)	160 (75)	.13	26 (84)	49 (78)	.49
≥2	14 (17)	53 (25)		5 (16)	14 (22)	
**ECOG score**						
0–1	62 (87)	172 (84)	.54	25 (89)	53 (88)	.90
≥2	9 (13)	32 (16)		3 (11)	7 (12)	
**Size of largest tumor**						
<5cm	33 (58)	113 (61)	.70	9 (39)	29 (53)	.27
≥5cm	24 (42)	73 (39)		14 (61)	26 (47)	
**IPI risk group**						
0–2	58 (70)	128 (59)	.082	20 (65)	37 (59)	.59
3–5	25 (30)	89 (41)		11 (35)	26 (41)	
**Therapy response**						
CR	74 (81)	178 (80)	.82	17 (53)	43 (67)	.18
PR	10	25		5	14	
SD	2	6		4	2	
PD	5	13		6	5	
**Ki-67**						
<70%	39 (43)	77 (35)	.20	6 (19)	19 (30)	.33
≥70%	52 (57)	143 (65)		26 (81)	44 (70)	
**Cell-of-origin**						
ABC	49 (54)	115 (52)	.80	15 (47)	22 (34)	.27
GCB	42 (46)	107 (48)		17 (53)	42 (66)	
**Nuclear p50**						
−	31 (34)	109 (50)	**.012**	13 (41)	38 (61)	.08
+	59 (66)	108 (50)		19 (59)	24 (39)	
**Nuclear p52**						
−	53 (59)	157 (76)	**.0053**	16 (50)	49 (83)	**.0014**
+	37 (41)	50 (24)		16 (50)	10 (17)	
**Nuclear p65**						
−	37 (41)	101 (47)	.38	10 (31)	20 (32)	1.0
+	54 (59)	115 (53)		22 (69)	42 (68)	
**Nuclear RelB**						
−	73 (81)	179 (86)	.30	24 (77)	55 (93)	**.043**
+	17 (19)	29 (14)		7 (23)	4 (7)	
***MYC* translocation**						
−	69 (93)	130 (87)	.18	19 (91)	30 (83)	.70
+	5 (7)	20 (13)		2 (9)	6 (17)	

**Abbreviations**: DLBCL, diffuse large B-cell lymphoma; LDH, lactate dehydrogenase; IPI, international prognostic index; CR, complete remission; PR, partial response; SD, stable disease; PD, progressive disease; GCB, germinal center B-like; ABC, activated B-cell-like.

#### Multivariate survival analysis in overall-, GCB- and ABC-DLBCL and in the WT-/MUT-p53 subsets

Multivariate survival analysis adjusting clinical parameters only (IPI alone or using individual five IPI components, sex, B-symptoms, and tumor size) indicated that c-Rel^+^ was not an independent prognostic factor. However, when the potentially compounding biomarkers (Myc^+^, Bcl-2^+^, and *TP53* mutations, Table 1) were also included in the multivariate analysis, c-Rel^+^ showed significant prognostic value for poorer overall survival in the whole and ABC-DLBCL cohorts. c-Rel^+^ also predicted poorer progression-free survival in ABC-DLBCL with a borderline *P* value (Table 3). c-Rel^+^ did not predict survival in GCB-DLBCL.

**Table 3:** Multivariate survival analysis of clinicopathologic parameters in DLBCLs treated with R-CHOP

Variables	OS			PFS		
	HR	95% CI	*P*	HR	95% CI	*P*
**Overall DLBCL**						
B-symptoms	1.54	1.06–2.23	**.024**	1.40	.97–2.01	.07
IPI >2	2.25	1.53–3.27	**<.0001**	1.96	1.36–2.81	**<.0001**
Female	.96	.66–1.41	.85	1.00	.70–1.43	.98
Tumor size ≥5cm	1.35	.94–1.95	.11	1.32	.93–1.87	.12
c-Rel^+^	1.55	1.08–2.23	**.018**	1.30	.92–1.83	.14
Bcl-2^+^	2.15	1.49–3.72	**<.0001**	1.92	1.15–2.72	**<.0001**
Myc^+^	2.35	1.49–3.72	**<.0001**	2.17	1.43–3.31	**<.0001**
*TP53* mutation	1.69	1.13–2.50	**.011**	1.69	1.36–2.81	**.007**
**ABC DLBCL**						
B-symptoms	1.29	.79–2.12	.31	1.23	.76–1.99	.39
IPI >2	2.17	1.29–3.67	**.004**	1.93	1.17–3.17	**.01**
Female	1.25	.77–2.03	.38	1.28	.80–2.05	.31
Tumor size ≥5cm	1.19	.74–1.92	.48	1.12	.71–1.77	.63
c-Rel^+^	1.69	1.06–2.68	**.026**	1.49	.96–2.33	.076
Bcl-2^+^	1.93	1.15–3.24	**.013**	1.85	1.13–3.00	**.014**
Myc^+^	2.18	1.16–4.09	**.015**	1.79	1.01–3.18	**.047**
*TP53*mutation	2.19	1.20–3.99	**.011**	1.88	1.04–3.39	**.035**
**DLBCL with WT-p53**						
B-symptoms	1.60	1.04–2.47	**.033**	1.55	1.03–2.35	**.037**
IPI >2	2.38	1.55–3.66	**<.0001**	2.13	1.42–3.18	**<.0001**
Female	.98	.63–1.52	.92	.96	.64–1.46	.86
Tumor size ≥5cm	1.22	.80–1.87	.36	1.10	.73–1.65	.65
c-Rel^+^	1.87	1.23–2.84	**.003**	1.58	1.07–2.34	**.023**
**DLBCL with MUT-p53**						
B-symptoms	1.21	.56–2.62	.63	1.06	.51–2.19	.87
IPI >2	2.91	1.40–6.04	**.004**	2.54	1.28–5.04	**.008**
Female	.77	.38–1.56	.47	.88	.45–1.71	.70
Tumor size ≥5cm	1.74	.84–3.62	.14	1.91	.96–3.79	.065
c-Rel^+^	.60	.27–1.34	.22	.62	.30–1.30	.21

**Abbreviations**: OS, overall survival; PFS, progression-free survival; HR, hazard ratio; CI, confidence interval; IPI, international prognostic index. Cutoffs for c-Rel^+^, Bcl-2^+^, and Myc^+^: 5% and 70% respectively.

Interestingly, in the WT-p53 subset, multivariate survival analysis adjusting clinical parameters indicated that c-Rel^+^ was an independent adverse prognostic factor (Table 3). Dividing into GCB and ABC subcohorts, the *P* values for the prognostic significance of c-Rel positivity remained significant for OS in both GCB- and ABC-DLBCL with WT-p53 after adjusting all the clinical parameters (*P* = 0.025 and *P* = 0.019 respectively), and for PFS in ABC-DLBCL with WT-p53 (*P* = 0.04). In the MUT-p53 subset, on the contrary, multivariate survival analysis adjusting clinical parameters suggested that the predictive value of c-Rel^+^ for poorer survival was not significant.

#### Potential molecular mechanisms underlying the prognostic impact of c-Rel positivity

A multitude of correlation and GEP analysis were performed to understand the prognostic effect of c-Rel nuclear expression observed in our cohorts.

#### Decreased AKT, Myc, and p53 expression in c-Rel^+^ patients

**Figure 3:**
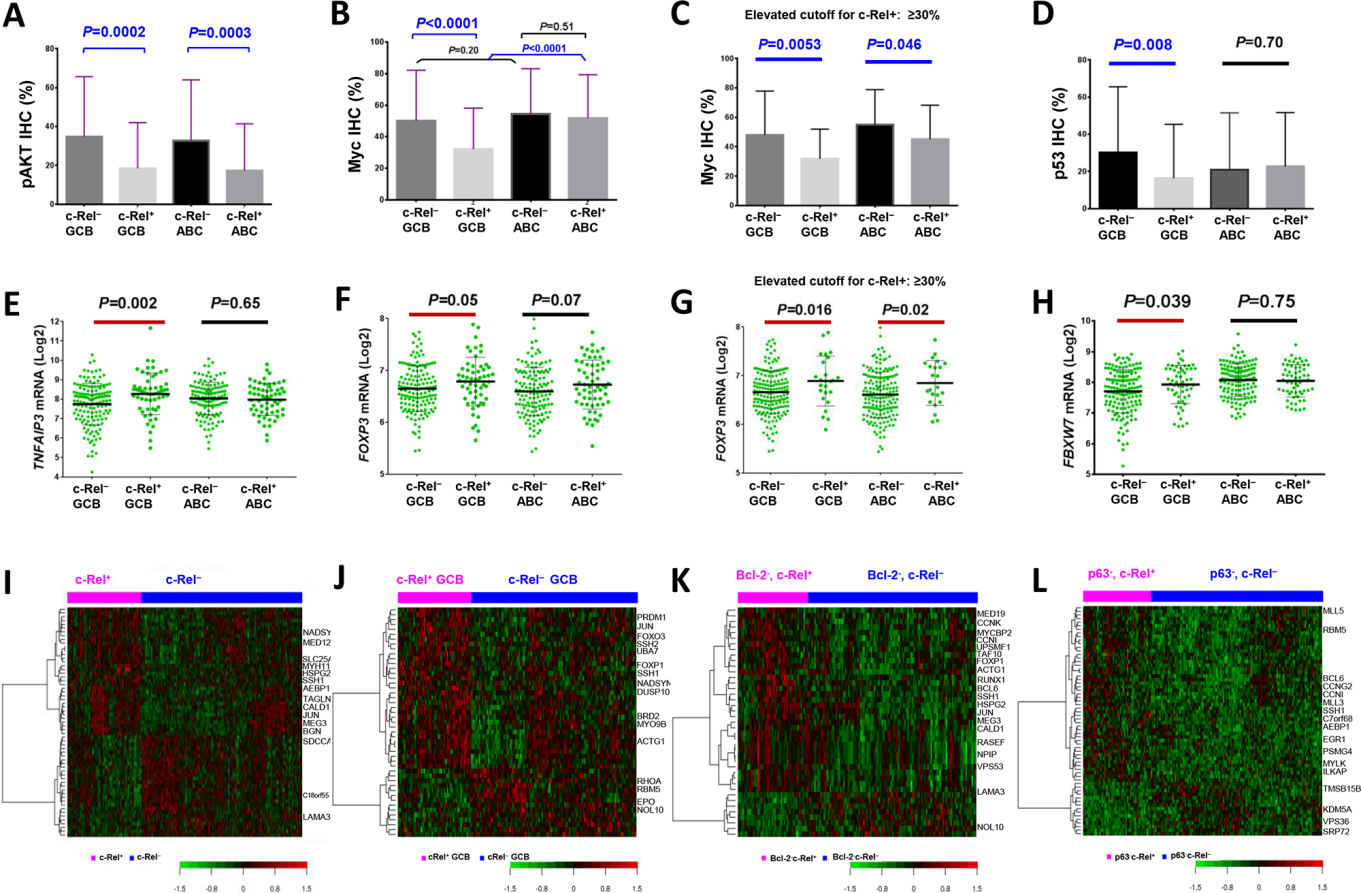
Gene and protein expression analysis correlating with c-Rel nuclear expression **A–D.** c-Rel positivity correlated with significantly lower levels of pAKT, Myc or p53 protein expression in DLBCL or GCB-DLBCL. **E–F.** *A20/TNFAIP3* which negatively regulates BCR, TNF, and NF-κB signaling, and c-Rel target gene *FOXP3*, were significantly upregulated in c-Rel^+^ GCB-DLBCL. **G.** Higher c-Rel expression levels (≥30%) correlated with significantly higher *FOXP3* mRNA levels in both GCB- and ABC-DLBCL. **H.** *FBXW7* was significantly upregulated in c-Rel^+^ GCB-DLBCL. Note: red lines indicate upregulation whereas blue lines indicated downregulation with significant or border-line *P* values. **I–J.** Heatmaps by gene expression profiling analysis between c-Rel^+^ and c-Rel^−^ DLBCL in the overall and GCB-DLBCL cohorts. **K.** Heatmap by gene expression profiling analysis between c-Rel^+^ and c-Rel^−^ DLBCL with low Bcl-2 expression (<70%). **L.** Heatmap by gene expression profiling analysis between c-Rel^+^ and c-Rel^−^ DLBCL without p63 expression.

Contrary to expectation at the protein level, there were inverse correlations between c-Rel and pAKT (*r* = −0.22, *P* = 0.0008; Fig. 3A, Supplementary Fig. S1F) in DLBCL, between c-Rel and Myc (*r* = −0.20, *P* = 2.92E-5), and between c-Rel and p53 (WT or MUT) (*r* = −0.20, *P* = 0.005) in GCB subtype by Spearman rank correlation (Fig. 3B, 3D; Supplementary Fig. S1G-S1H). Higher c-Rel expression levels (≥30%) coincided with decreased Myc in both GCB- and ABC-DLBCL (Fig. 3C). These correlations may not have resulted from transcriptional regulations by c-Rel, since c-Rel positivity did not correlate with *AKT1, MYC* (Supplementary Fig. S1I-1J) or *TP53* (*P* = 0.34 in GCB-DLBCL, and *P* = 0.088 for *TP53* upregulation in ABC-DLBCL) mRNA expression significantly, and that in GCB-DLBCL cases without *MYC* translocations, c-Rel^+^ still correlated with decreased Myc levels in GCB-DLBCL.

To understand the mechanisms underlying these inverse correlations, we compared the mRNA expression levels of genes known for c-Rel activation in c-Rel^+^ and c-Rel^−^ DLBCL. We found that genes involved in BCR signaling, including *CD79A, CD19, LYN, SYK, CARD11, MALT1, BLNK, BTK*, and *ZAP70* [2], and *MAP3K7*/*TAK1* [7], were significantly upregulated in c-Rel^+^ compared with c-Rel^−^ GCB-DLBCL. These genes did not show significantly differential expression correlating to c-Rel^+^ in ABC-DLBCL (Supplementary Fig. S3A-S3J). TNF, MAPK and TLR signaling which are also known as NF-κB activation mechanisms [3, 11], might not contribute to c-Rel activation significantly in our DLBCL cohort, suggested by non-significant correlations between c-Rel positivity and gene expression of *CD40* (GCB, *P* = 0.23; ABC, *P* = 0.70), *MAP3K14*/*NIK* (GCB, *P* = 0.39; ABC, *P* = 0.29), *BAFF* (GCB, *P* = 0.38; ABC, *P* = 0.73), *TNF* (GCB, *P* = 0.51; ABC, *P* = 0.61), *TNFRSF11* (GCB, *P* = 0.75; ABC, *P* = 0.83), *TNFSF11* (GCB, *P* = 0.74; ABC, *P* = 0.84), *TNFRSF8* (GCB, *P* = 0.51; ABC, *P* = 0.22), *TNFSF8* (GCB, *P* = 0.69; ABC, *P* = 0.72), *TRAIL* (GCB, *P* = 0.58; ABC, *P* = 0.17), *TRAF1*/2/5/6 (GCB, *P* = 0.10, 0.89. 0.48 and 0.43 respectively; ABC, *P* = 0.37, 0.66, 0.35 and 0.83 respectively), *TANK* (GCB, *P* = 0.37; ABC, *P* = 0.44), *MAP3K8* (GCB, *P* = 0.12; ABC, *P* = 0.51), *MAP3K3* (GCB, *P* = 0.89; ABC, *P* = 0.68), *TLR4* (GCB, *P* = 0.53; ABC, *P* = 0.06 for downregulation) and *etc*. However, *TNFRSF13C* (encoding BAFFR) and *TLR2* were significantly upregulated in GCB-DLBCL (*P* = 0.017 and *P* = 0.018 respectively). Other *TLR*s (*TLR*1, 3, 5–10) did not show significantly differential expression between the c-Rel^+^ and c-Rel^−^ groups.

On the other hand, *A20/TNFAIP3* and *TNIP1* which terminate NF-κB signaling [37] were also significantly upregulated in c-Rel^+^ GCB-DLBCL (Fig. 3E, Supplementary Fig. S3K). Analyzing expression of NF-κB regulators IKK and IκB genes showed that *IKK2/IKBKB* (but not *IKK1*) was upregulated in c-Rel^+^ GCB-DLBCL (marginal *P* value; Supplementary Fig. S3L), suggesting that activation of c-Rel was mediated through the canonical pathway [2]. Genes encoding IκBα/β/ε (which sequester NF-κB in the cytoplasm) and IκB-zeta (which inhibits NF-κB transcription activity) were significantly upregulated in either GCB- or ABC-DLBCL (Supplementary Fig. S3M-P), resembling p65 function [38].

Upregulation of *TNFAIP3*/*A20* and *TNIP1* (which inhibit MALT1, IKK3 and TRAF6 [37]) in c-Rel^+^ GCB-DLBCL (Fig. 3E) may be relevant for the decrease of pAKT and Myc levels coincided with c-Rel positivity (Fig. 3A-3C) and the lack of c-Rel prognostic impact [39, 40]. Upregulation of *IKK2* and *IκB*s in c-Rel^+^ GCB-DLBCL may contribute to the decreased p53 levels in GCB-DLBCL (Fig. 3D) [41]. In addition, decrease of Myc may also result from *MYC* repression by FOXP3 [42] and posttranslational regulation of Myc stability by the ubiquitin-proteasome system. This was suggested by the upregulation of *FOXP3* (Fig. 3F-3G) in c-Rel^+^ DLBCL which was opposite to the decrease of Myc in c-Rel^+^ DLBCL (≥5% and 30% cutoff respectively) (Fig. 3B-3C), and upregulation of *FBXW7* (Fig. 3H), *PIN1* (*P* < 0.0001), and *PPP2R2A* (*P* = 0.043) (which facilitate Myc degradation [43]) in c-Rel^+^ GCB-DLBCL.

#### Expression of c-Rel target genes

c-Rel is known to transcriptionally regulate genes involved in inflammation, immune cell development and cell survival [4, 7]. Gene expression analysis between c-Rel^+^ and c-Rel^−^ DLBCL showed up- or downregulation of *FOXP3* (Fig. 3F-3G), *IL1B, IL3, IL6, IL10RA, IL12B, IL12RB1, IL17A, STAT3, JAK1/3, RUNX1/3, CXCR4, PRDM1, TP63*, and *CDKN1A* (border-line *P* value) (Supplementary Fig. S4B-O) in c-Rel^+^ *versus* c-Rel^−^ DLBCL, either in the GCB or ABC subtype. c-Rel did not appear to correlate with transcription of apoptotic genes significantly (data not shown) except antiapoptotic *CFLAR* (upregulated in GCB-DLBCL, *P* = 0.043, Supplementary Fig. S4P). Gene expression of antiapoptotic *BCL2L1* (*P* = 0.13), *MCL1* (*P* = 0.10), and *TRAF1* (*P* = 0.10) also tended to be higher in c-Rel^+^ GCB-DLBCL.

#### Gene expression signature of c-Rel expression in overall-, GCB- and ABC-DLBCL

To better understand regulation and function of c-Rel underlying its clinical impact, genome-wide gene expression of c-Rel^+^ and c-Rel^−^ DLBCLs were compared in the overall and subsets of cohorts. Distinct GEP signatures were shown in overall- (Fig. 3I; Table 4) and GCB- (Fig. 3J; Supplementary Table S1) but not in ABC-DLBCL. These c-Rel signatures showed similarity and difference with that in T-cells [44], including genes involved in signaling, transcription, differentiation, tumor suppression, metabolism, cytoskeleton, adhesion, extracellular matrix assembly, metastasis and angiogenesis. *AEBP1* promoting degradation of IκBα and NF-κB activation, and *UBA7* encoding an E1 ubiquitin-activating enzyme were upregulated, whereas *SDCCAG1* with a role in nuclear export was downregulated in c-Rel^+^ DLBCL. *BRD2* encoding a BET transcription factor, which enhances IKK activity and NF-κB activation in ABC-DLBCL *in vitro* and *in vivo* [45], was significantly upregulated in c-Rel^+^ GCB-DLBCL. Upregulation of *FOXO3* in c-Rel^+^ GCB-DLBCL, which is negatively regulated by PI3K/AKT and inhibits MYC expression and function directly or indirectly [46], is consistent with the decreased pAKT and Myc levels in c-Rel^+^ DLBCL. *MEG3*, which encodes a long non-coding RNA that increases p53 levels, and tumor suppressor gene *RBM5* were upregulated in c-Rel^+^ DLBCL and c-Rel^+^ GCB-DLBCL respectively.

**Table 4:** Gene signatures of c-Rel^+^ in the overall DLBCL cohort (false discovery rate < .10), and gene signatures of *REL* amplification identified in the overall (false discovery rate < .05, fold change >2) or GCB-DLBCL cohort (false discovery rate < .05)

Function	c-*Rel*^+^ *vs.* c-*Rel*^−^		*REL* amp^+^ *vs. REL* amp^−^	
	Upregulated	Downregulated	Upregulated	Downregulated
**Mitogen, cytokine, growth factor, receptors, signal transduction, NF-κB activation**	*CTGF, AEBP1, IGFBP7, GPR124, RASEF, PTGFRN*		PTP4A3, CCL17, KISS1R*, IL8*, CD80*, CAMKK2*	*P2RX5, IL7, PRKCB*
**DNA replication, recombination, cell cycle**	*MLF1IP*	*C10orf78*	STAG3, H1F0*, NDNL2*	
**Gene expression, transcription and translation regulation**	*JUN, MED12, SFMBT2, NSD1*	*KDM5A, ZNF267, TCERG1*	*REL*^‡^, *PUS10*^‡^, *FYTTD1, CREM*^‡^, *DENND4A*^‡^, *ZNF711, KDM4B, PAPOLG*^‡^, SSX4, MED13L*	*FOXP1*
**Actin, cytoskeleton, collagen, cell morphology, adhesion, extracellular matrix, migration, muscular system function**	*TAGLN, CALD1, MYH11, CCDC80, SSH1, BGN, HSPG2, KIAA1109*	*LAMA3*	*CCT4, PLS3, KIF26B*^‡^, ABLIM1, DYNC1I1*, DNAH14*	
**Protein sorting, protein and vesicle's trafficking, transportation, chaperone**	*GGA3, COG5*	*SEC23B, PLDN, TXNDC9, TGOLN2, SRP72, SDCCAG1, NIPSNAP3A*	*CSMD1*^‡^, *CLCNKB, AHSA2*^‡^, *XPO1, PEX13*^‡^	
**Metabolism, redox**	*NADSYN1, CKMT1A/B, SLC25A16, POMT2*	*C18orf55, YME1L1*	*CTPS2*	ODC1, GBA2*
**Tumor suppressor, apoptosis, autophagy**	*MEG3*	*CISD2*	*CSMD1*^‡^, *TUSC1, C20orf117*	
**Degradation**			*RNF180, USP34*^‡^, MAGEA3, COMMD1*	
**Unknown function**	*NPIP, ANO8*	*JRKL, DNAJC9, GCOM1/GRINL1A, NOL10*	*CT45A5*^‡^, *MAGEA9*^‡^, *BTNL9, SYT17, KIAA1841, DNAJC5B, FAM9C*^‡^, *CTAG1A/B*^‡^, ZCCHC7, ZC3HAV1L*, LOC339803*, ACOXL*, C22orf37*, PRUNE2*, DUSP5P*, CENPBD1*, MAGEA5*, MEGF8*	*MPEG1*

* Genes identified in the comparison of patients with and without *REL* amplification within GCB-DLBCL cohort only;

‡ Genes identified in both comparisons (comparisons in the overall cohort and in the GCB-DLBCL cohort).

#### Functional dependence on other NF-κB subunits and distinctive function of c-Rel in overall-, GCB- and ABC-DLBCL

In order to identify the dependency and distinctive functions of c-Rel *versus* other NF-κB subunits, we compared the GEP of c-Rel^+^ and c-Rel^−^ within p50^−^, p65^−^, p52^−^, RelB^−^, p50^+^, p65^+^ p52^+^ and RelB^+^ DLBCL subsets individually. c-Rel showed GEP signatures in p50^+^, p65^+^, p52^−^ and RelB^−^ DLBCL subsets (Supplementary Fig. S2M-2N; Supplementary Table S2), but not in p50^−^, p65^−^, p52^+^ or RelB^+^ DLBCL. These results may suggest that c-Rel functions mainly through the canonical pathway in the forms of c-Rel/p50 and c-Rel/p65 dimers. The results also imply that c-Rel/p65 and c-Rel/p50 dimers have significantly distinctive activities compared to other p65 or p50 dimers (mainly p50/p65 and p50/p50 dimers [22, 47], potentially also p65/p65 [48], p50/p52 [49], p50/RelB dimers [22]).

c-Rel function in GCB-DLBCL did not appear to depend on other single NF-κB members significantly, because no c-Rel signatures were identified within p50^+^, p65^+^ p52^+^ or RelB^+^ GCB-DLBCL, whereas 16 differentially expressed genes (DEGs) were identified within the RelB^−^ GCB-DLBCL subset by a high false discovery rate (FDR < 0.30) threshold.

Although c-Rel nuclear expression did not show distinctive GEP signature in the overall ABC-DLBCL, within the p65^+^ ABC-DLBCL subset there were 64 DEGs between c-Rel^+^ and c-Rel^−^ (FDR < 0.25), and within the p50^+^ ABC-DLBCL subset 28 DEGs (FDR < 0.30) (Supplementary Fig. S2O-2P; Supplementary Table S3), supporting the idea that c-Rel function depended on p65 and p50 activation as suggested by the survival analysis (Fig. 2E-2F). On the other hand, the differential expression of these DEGs between c-Rel^+^ and c-Rel^−^ within the p65^+^ or p50^+^ ABC-DLBCL groups also suggested that c-Rel/NF-κB dimers compared with other p65 or p50 dimers have significantly distinct roles in regulation of proliferation, apoptosis, metabolism, trafficking, cell adhesion, migration, and angiogenesis. In c-Rel^+^/p65^+^ (*versus* c-Rel^−^/p65^+^) ABC-DLBCL, *SIGIRR* which attenuates the TLR4 signaling, *AEBP1, TFE3* (which activates CD40L expression), and *HSPB1* (encoding Hsp27 which can either decrease IKK2 activity [50], or enhance proteasomal degradation of IκBα [51]), were upregulated; *SETD6*, encoding a methyltransferase which impedes p65 function, was downregulated. In both c-Rel^+^/p65^+^ (*versus* c-Rel^−^/p65^+^) and c-Rel^+^/p50^+^ (*versus* c-Rel^−^/p50^+^) ABC-DLBCL, *PSMG1* which promotes assembly of the 20S proteasome was downregulated.

#### c-Rel signatures in Bcl-2^−^ and p63^−^ DLBCL

To understand the significant prognostic impact of c-Rel expression in Bcl-2^−^ and p63^−^ DLBCL (Fig. 2D, 2L), GEP analysis was also performed in Bcl-2^−^ and p63^−^ DLBCL subsets. In Bcl-2^−^ DLBCL, c-Rel expression was associated with upregulation of *BCL6* (required for GC formation), *JUN, MYCBP2* (MYC binding protein 2, involved in Myc transcriptional activities and degradation of target proteins), cyclin genes *CCNK* and *CCNI, LPIN1* involved in metabolism, *DDR2* encoding a tyrosine kinase, and *PSMF1*, which inhibits the hydrolysis of protein and peptide substrates by the 20S proteasome (Fig. 3K). In p63^−^ DLBCL, c-Rel expression was associated with upregulation of *BCL6, EGR1, AEBP1, C7orf68, CCNG2, CCNI, ILKAP*, and *PSMG4* (encoding a chaperone protein which promotes assembly of the 20S proteasome) and downregulation of *FBXO22* (involved in degradation of specific proteins in response to p53 induction) (Fig. 3L). In contrast, no genes were significantly differentially expressed between c-Rel^+^ and c-Rel^−^ patients in the Bcl-2^+^ or p63^+^ DLBCL subset. To understand the tumor suppressor function of p63 towards c-Rel signaling, we further compared GEP between p63^+^ and p63^−^ patients within the c-Rel^+^ DLBCL subset, and found that *LYN* was significantly downregulated in p63^+^ DLBCL, suggesting that p63 may inhibit BCR signaling thus attenuate c-Rel activation.

#### c-Rel signatures in the WT-p53 and MUT-p53 subsets and crosstalk between c-Rel and the p53 Pathway

c-Rel nuclear expression showed distinctive GEP signature only in the WT-p53 subcohort, (Supplementary Fig. S2L), but not in the MUT-p53 subcohort probably due to the heterogeneous and dominant MUT-p53 function [36]. The c-Rel GEP signature in the WT-p53 subcohort included oncogene *JUN, CTTN* which contributes to tumor cell invasion and metastasis, *ENG* involved in the regulation of angiogenesis, *SH3GL1* with a role in cell cycle whose overexpression may play a role in leukemogenesis, *LPIN1* and *CKMT1A/B* involved in metabolism regulation, and many genes involved in Golgi function. On the other hand, *RASEF* with a potential role as tumor suppressor, and *CREBZF* (a positive regulator of p53 [52]) were also upregulated, whereas *YME1L1* which plays a role in mitochondrial protein metabolism and promotes antiapoptotic activities was downregulated. DEGs involved in epigenetic regulation include upregulatd *NSD1* and downregulated *KDM5A* and *MYSM1* (Supplementary Table S4).

**Figure 4:**
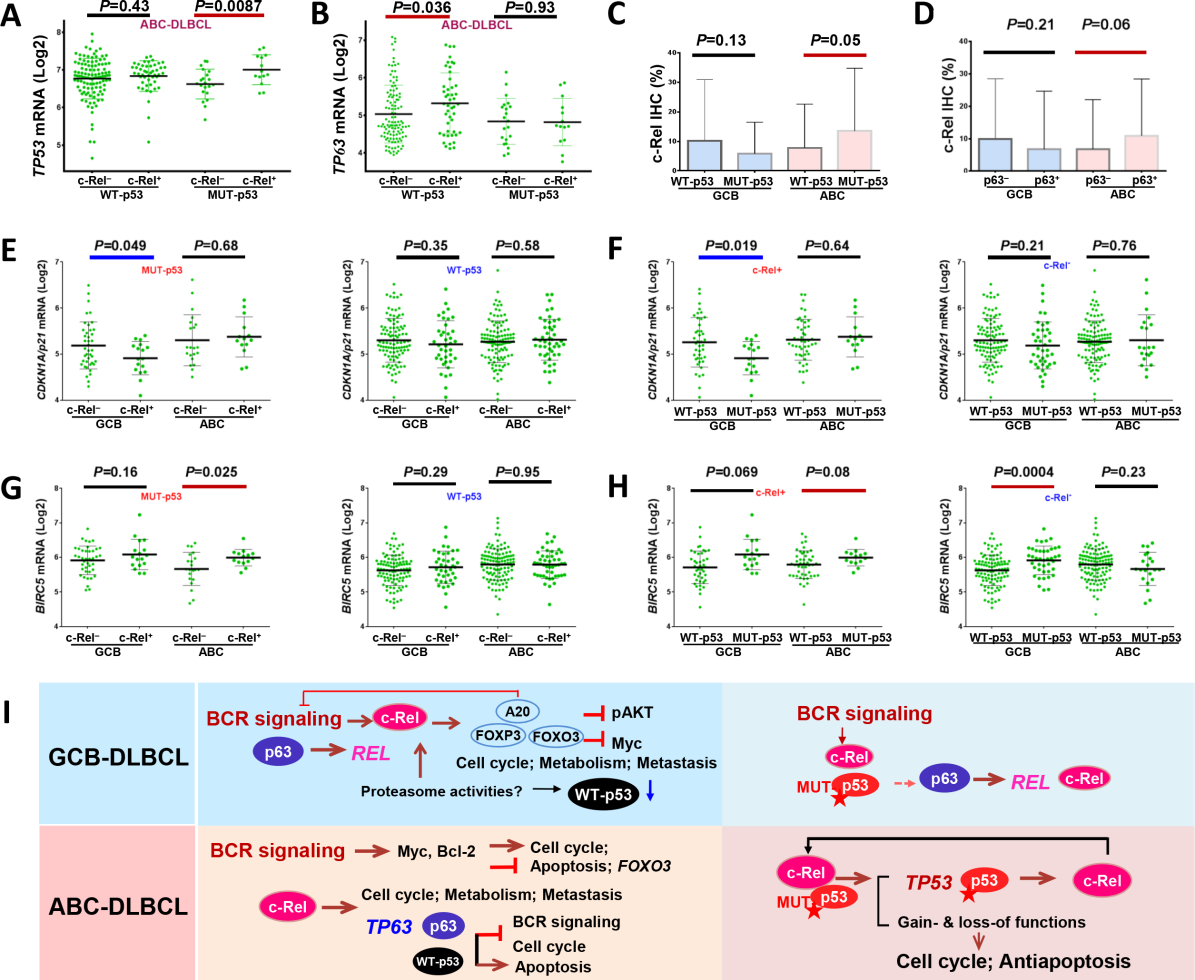
Crosstalk between c-Rel and the p53 pathway **A.** In ABC-DLBCL with MUT-p53, c-Rel nuclear expression was associated with significantly higher *TP53* mRNA. **B.** In ABC-DLBCL with WT-p53, c-Rel nuclear expression was associated with significantly higher *TP63* mRNA. **C.** In ABC-DLBCL, *TP53* mutations were associated with significantly higher c-Rel nuclear expression levels. **D.** In ABC-DLBCL, p63 expression coincided with higher c-Rel nuclear expression levels. **E.** c-Rel nuclear expression significantly correlated with *CDKN1A/p21* downregulation in GCB-DLBCL with MUT-p53, but not in GCB-DLBCL with WT-p53. **F.** *TP53* mutations significantly correlated with *CDKN1A/p21* downregulation in GCB-DLBCL with c-Rel nuclear expression, but not in GCB-DLBCL without c-Rel nuclear expression. **G.** c-Rel nuclear expression significantly correlated with *BIRC5* upregulation in ABC-DLBCL with MUT-p53, but not in ABC-DLBCL with WT-p53. **H.** In ABC-DLBCL with c-Rel nuclear expression, *TP53* mutations appeared to be associated with higher *BIRC5* transcription (marginal *P* value); in contrast without c-Rel nuclear expression, p53 mutant group correlated with significantly higher *BIRC5* transcription in GCB-DLBCL, whereas appeared to have slightly lower *BIRC5* transcription in ABC-DLBCL. Note: red lines indicate upregulation with significant or border-line *P* values whereas blue lines indicated downregulation. **I.** Hypothetical models of crosstalk between c-Rel, p53, and p63 in GCB- and ABC-DLBCL with WT- or MUT-p53 suggested by our data.

Moreover, nuclear c-Rel positivity coincided with significantly upregulated *TP53* transcription in ABC-DLBCL with MUT-p53 (Fig. 4A), whereas significantly upregulated *TP63* and p63 protein levels in ABC-DLBCL with WT-p53 (Fig. 4B, Supplementary Fig. S5A). Conversely, in ABC-DLBCL, *TP53* mutations were associated with significantly upregulated *REL* mRNA (Supplementary Fig. S5B) and higher nuclear c-Rel protein levels (Fig. 4C), and p63+ ABC-DLBCL had trend for elevated nuclear c-Rel protein levels (*P* = 0.06, Fig. 4D). In GCB-DLBCL, expression of MUT-p53 and p63 was associated with higher *REL* mRNA (*P* = 0.082 and *P* = 0.0016 respectively, Supplementary Fig. S5C-S5D). To exclude *REL* amplification as a potential compounding factor, we performed the same analyses in patients without *REL* amplification or polysomies, and found that only the correlation between *TP53* mutations (but not p63^+^) and upregulated *REL* mRNA (but not the c-Rel protein) in ABC-DLBCL was affected.

c-Rel^+^ DLBCL with MUT-p53 which was associated with significantly worse survival (Fig. 2H-2I), also correlated with decreased pAKT and Myc expression (Supplementary Fig. S2I-2J), as seen in c-Rel^+^ DLBCL with WT-p53 which did not show worse survival by univariate survival analysis (Fig. 2G). Therefore to confer worse prognostic impact, c-Rel must have used other oncogenic pathways. We analyzed expression of c-Rel and p53 target genes, which appeared to suggest that MUT-p53 and c-Rel gained functions in downregulating *p21* in GCB-DLBCL (Fig. 4E-4F), and upregulating *BIRC5* (encoding antiapoptotic survivin) in ABC-DLBCL (Fig. 4G-4H). Our data also suggested possible lost-of-function of c-Rel in the presence of MUT-p53 in upregulating *TP63* in ABC-DLBCL, and gain-of-function in inducing *NFKB1, TANK*, and *BCL2L11* in GCB-DLBCL, *AURKB, RELA*, and *BAD* in ABC-DLBCL, as well as downregulating *TRAF2* in GCB-DLBCL and *BCL2L11* in ABC-DLBCL (Supplementary Fig. S5–S6).

A hypothetical model for the reciprocal induction of *REL, TP53* and *TP63* and other biology suggested by GEP analysis in GCB- and ABC-DLBCL with WT- or MUT-p53 is depicted in Fig. 4I.

### Clinical relevance and gene expression signature of *REL* amplification in DLBCL

**Figure 5:**
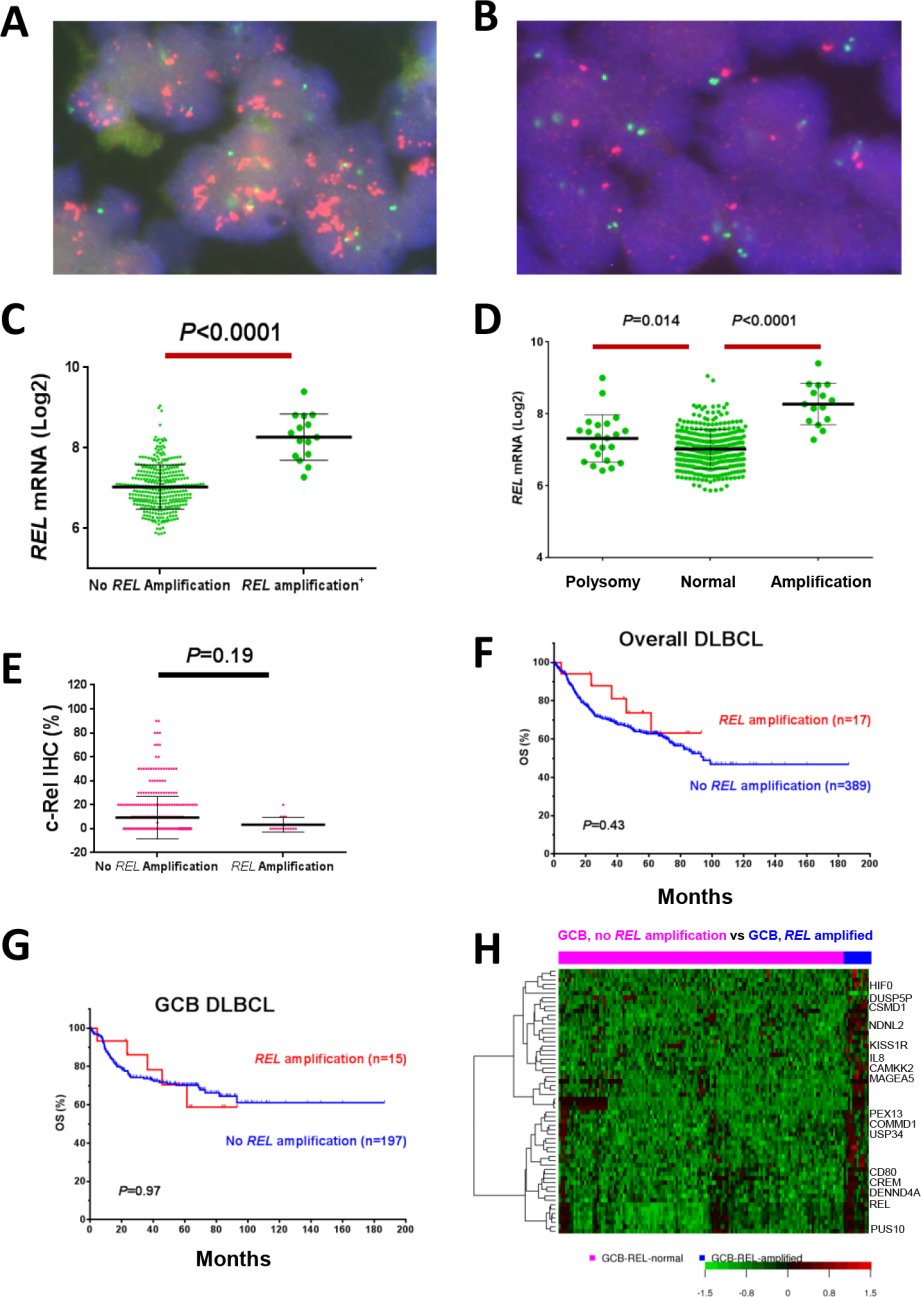
REL amplification analysis in DBLCL **A–B.** Representative DLBCL cases positive or negative for *REL* amplification by fluorescence *in situ* hybridization analysis. **C–D.** *REL* amplification correlated with significantly higher *REL* mRNA levels. **E.** *REL* amplification did not correlate with c-Rel nuclear expression levels. **F–G.** *REL* amplification did not correlate with patient survival in overall- or GCB-DLBCL. **H.** Heatmap of gene expression profiling analysis for *REL* amplification in GCB-DLBCL.

*REL* amplification detected by FISH (Fig. 5A-5B) was found predominantly in GCB-DLBCL (only two cases of ABC-DLBCL), with a frequency of 4.2% of overall DLBCL, or 7.1% of GCB-DLBCL. *REL* amplification correlated with significantly higher *REL* mRNA levels (Fig. 5C-5D), but not with c-Rel nuclear expression (Fig. 5E), clinical parameters (Table 5) or patient survival either in overall- or GCB-DLBCL (Fig. 5F-5G), suggesting the importance of posttranslational regulations for c-Rel activation and function.

**Table 5:** Clinicopathologic characteristics of 407 *de novo* DLBCL patients tested for *REL* amplification status

Variables	DLBCL		*P*	GCB-DLBCL		*P*
	*REL* amp^+^	*REL* amp^−^		*REL* amp^+^	*REL* amp^−^	
	N (%)	N (%)		N (%)	N (%)	
**Patients**	17(100)	390(100)		15(100)	198(100)	
**Gender**						
Male	7 (41)	239 (61)	.097	6 (40)	120 (61)	.17
Female	10 (59)	151 (39)		9 (60)	78 (39)	
**Age (yr)**						
<60	8 (47)	170 (44)	.77	8 (53)	103 (52)	1.0
≥60	9 (53)	220 (56)		7 (47)	95 (48)	
**Stage**						
I-II	10 (59)	180 (48)	.37	9 (60)	107 (56)	.75
III-IV	7 (41)	198 (52)		6 (40)	85 (44)	
**B-symptoms**						
No	13 (77)	242 (65)	.35	12 (80)	130 (70)	.42
Yes	4 (23)	128 (35)		3 (20)	55 (30)	
**Serum LDH**						
Normal	8 (47)	144 (41)	.60	7 (47)	77 (43)	.81
Elevated	9 (53)	210 (59)		8 (53)	100 (57)	
**# of extranodal sites**						
0-1	16 (94)	287 (76)	.085	14 (93)	148 (78)	.17
≥2	1 (6)	90 (24)		1 (6)	41 (22)	
**Performance score**						
0-1	17 (100)	289 (84)	.068	15 (100)	145 (85)	.11
≥2	0 (0)	59 (16)		0 (0)	25 (15)	
**Size of largest tumor**						
<5cm	8 (57)	176 (59)	.91	7 (54)	90 (61)	.62
≥5cm	6 (43)	124 (41)		6 (46)	58 (39)	
**IPI risk group**						
0–2	14 (82)	239 (89)	.11	13 (87)	130 (68)	.14
3–5	3 (18)	139 (11)		2 (13)	62 (32)	
**Therapy response**						
CR	14 (82)	295 (76)	.77	12 (80)	146 (74)	1.0
PR	0	51		0	24	
SD	0	16		0	10	
PD	3	28		3	18	
**Cell-of-origin**						
ABC	2 (12)	190 (49)	**.0024**	0 (0)	0 (0)	-
GCB	15 (88)	198 (51)		15 (100)	198 (100)	
**Primary origin**						
Nodal	9 (53)	253 (66)	.30	7 (53)	131 (68)	.10
Extranodal	8 (47)	130 (34)		8 (47)	63 (32)	
**Ki-67**						
<70%	5 (29)	147 (38)	.61	5 (33)	82 (42)	.59
≥70%	12 (71)	238 (62)		10 (68)	112 (58)	
***TP53* mutation**						
MUT *TP53*	6 (35)	79 (22)	.20	5 (33)	46 (25)	.49
WT *TP53*	11 (65)	280 (78)		10 (67)	136 (25)	
**p53 expression**						
+	8 (47)	126 (36)	.44	7 (47)	66 (37)	.58
−	9 (53)	227 (64)		8 (53)	113 (63)	
***MYC* translocation**						
+	1 (8)	31 (12)	1.0	1 (10)	21 (17)	1.0
−	11 (92)	228 (88)		9 (90)	102 (83)	
***BCL2* translocation**						
+	5 (29)	54 (16)	.18	5 (33)	45 (29)	.77
−	12 (71)	273 (84)		10 (67)	112 (71)	
***BCL6* translocation**						
+	5 (33)	88 (32)	.91	5 (38)	33 (24)	.24
−	10 (67)	187 (68)		8 (62)	106 (76)	
**Bcl-2 expression**						
+	5 (29)	191 (50)	.13	4 (27)	80 (41)	.41
−	12 (71)	194 (50)		11 (73)	115 (59)	
**Myc expression**						
+	5 (29)	117 (30.5)	1.0	4 (27)	55 (29)	1.0
−	12 (71)	266 (69.5)		11 (73)	137 (71)	
**pAKT**						
+	1 (6)	75 (20)	.21	1 (6)	37 (19)	.31
−	16 (94)	307 (80)		14 (94)	156 (81)	
**p16**						
+	10 (67)	95 (29)	**.0038**	9 (69)	58 (35)	**.017**
−	5 (87)	230 (71)		4 (31)	110 (65)	
**Nuclear p50**						
+	6 (40)	188 (52.7)	.43	6 (46)	78 (42)	.78
−	9 (60)	169 (47.3)		7 (54)	106 (58)	
**Nuclear p52**						
+	5 (33.3)	99 (27)	.56	4 (31)	56 (30)	1.0
−	10 (66.7)	268 (73)		9 (69)	129 (70)	
**Nuclear p65**						
+	8 (50)	223 (60.6)	.44	7 (50)	113 (61)	.41
−	8 (50)	145 (39.4)		7 (50)	72 (39)	
**Nuclear RelB**						
+	0 (0)	58 (15.9)	.14	0 (0)	27 (15)	.22
−	15 (100)	306 (84.1)		13 (100)	159 (85)	

**Abbreviations**: DLBCL, diffuse large B-cell lymphoma; GCB, germinal center B-cell like; ABC, activated B-cell like; LDH, lactate dehydrogenase; IPI, international prognostic index; CR, complete remission; PR, partial response; SD, stable disease; PD, progressive disease.

**Note**: Immunohistochemistry cutoff for biomarkers: c-Rel, p50, p52, p65, RelB, 5%; p53, ≥20%; Myc, ≥70%; Bcl-2, ≥70%; pAKT, ≥70%; p16, >10%.

*REL* amplification showed distinct GEP signatures in either overall or GCB-DLBCL (Table 4; Fig. 5H). Except *CCT4* gene which was also mapped to 2p as *REL*, these DEGs were not overlapping with those associated with 2p gain in chronic lymphocytic leukemia [53]. *USP34* (mapped to 2p; encoding a deubiquitinase which negatively regulates NF-κB activation), *COMMD1* (mapped to 2p; COMMD1 can enhance p65 nuclear degradation), *RNF180* (E3 ubiquitin-protein ligase), and *XPO1* (mapped to 2p; encoding CRM1 which enhances p65 nuclear export) were upregulated. Upregulated *MAGEA3*, which stimulates p53 ubiquitination by enhancing TRIM28 ubiquitin ligase activity, could negatively regulate p53 levels. However, proapoptotic *PUS10* (mapped to 2p) and the tumor suppressor genes *CSMD1, KISS1R, NDNL2, TUSC1* and *DENND4A* (repressing *MYC* transcription) were upregulated, which may also explain the lack of prognostic significance of *REL* amplification.

## DISCUSSION

c-Rel is a unique NF-κB member important for lymphocyte development, proliferation and survival [4, 17], however, the clinical relevance of c-Rel activities in DLBCL has not been well studied with inconsistent results. In a cohort of 460 DLBCL patients, we found c-Rel nuclear expression positive in 26% of DLBCL patients at lower levels than p65 and p50, and associated with extranodal DLBCL. c-Rel nuclear expression conferred adverse impact in ABC-DLBCL with context-dependent prognostic significance. Remarkably, c-Rel nuclear expression had significantly synergistic effects with *TP53* mutations. Although c-Rel positivity did not show prognostic significance in DLBCL with WT-p53, multivariate analysis indicated that c-Rel was an independent adverse prognostic factor after adjusting clinical parameters. Compared with studies in the literature, the positivity frequency in our study is lower than the 65% and 64% by two previous reports (0% and 30% cutoff respectively) [34, 35], and higher than the 18% by another study using *a* >50% cutoff [54]; the prognostic significance of c-Rel nuclear expression in MUT-p53 and various ABC-DLBCL subsets demonstrated in our cohort have not been reported previously. Moreover, we also found that *REL* amplifications in 4.2% of DLBCL had no correlation with nuclear accumulation of c-Rel (consistent with a previous study [33]) or prognosis (no earlier studies have been reported). In fact, if polysomy cases (46% are of ABC subtype) are also included into *REL* amplified cases which resulted in a frequency of 12% for *REL* amplification in DLBCL, *REL* amplification correlated with better patient survival in ABC- but not in GCB-DLBCL. We further found that several genes, which are also mapped to 2p, and involved in deubiquitination of IκB, degradation, nuclear export of NF-κB, or proapoptosis, were highly expressed in *REL* amplified cases likely due to co-amplification.

The lack of prognostic impact of c-Rel nuclear expression in GCB-DLBCL probably results from the decrease in Myc, AKT and p53 expression, and the complicated interaction and relationships with other NF-κB subunits. Upregulation of *FOXP3* [42], *FOXO3* [46], *A20* [39, 40], *IKK2* and *IκB*s [41, 49] in c-Rel^+^ GCB-DLBCL may be relevant for the reductions as well as the phosphorylation-dependent ubiquitin-proteasome system which mediates c-Rel activation and degradation of Myc and p53 [43, 55]. Decreased p53 and Myc levels in c-Rel^+^ GCB-DLBCL may be necessary for GC reaction [56] due to the proapoptotic function of p53 and Myc [22]. In addition, that c-Rel target FOXP3 in turn represses c-Rel activation and inhibits c-Rel function shown by an earlier study [57] may also explain the lack of prognostic effect of c-Rel expression in GCB-DLBCL. In contrast, in ABC-DLBCL, elevated IKKs and other activated signaling (such as BCR) may have increased Myc protein stability [40]; and overexpressed Myc in turn inhibits FOXO3 function [58].

In agreement with a recent study that demonstrated c-Rel is required for the GC maintenance [17], our data showed distinctive c-Rel signatures in GCB- DLBCL but not in the overall ABC-DLBCL cohort (Fig. 3J); *BCL6*, essential for GC maintenance, was upregulated in both c-Rel^+^/Bcl-2^−^ and c-Rel^+^/p63^−^ DLBCL (Fig. 3K-3L). c-Rel may have different functions by forming different NF-κB dimers. In ABC-DLBCL, c-Rel function depends on p50 and p65 suggested by GEP (Supplementary Fig. S2O-2P) and survival analysis (Fig. 2E-2F). Moreover, in Bcl-2^−^ DLBCL especially Bcl-2^−^ ABC-DLBCL, c-Rel^+^ correlated with significantly poorer survival (Fig. 2D), supporting the idea that c-Rel exerted its oncogenic function via Bcl-2-independent pathways [17].

We attempted to understand the dependence and differences between c-Rel and other NF-κB members. Coexpression in patients and coimmunoprecipitation analysis in primary DLBCL cells suggest c-Rel can form complexes with all NF-κB subunits. However, by GEP and survival analyses dissecting c-Rel function and prognostic impact with and without concurrent activation of other NF-κB subunits, our results suggested that the oncogenic c-Rel dimers with clinical significance are likely predominated of c-Rel/p65 and c-Rel/p50 in ABC-DLBCL, and potentially c-Rel/c-Rel dimers in GCB-DLBCL, which are all activated via the canonical pathway [4, 17, 68, 69].

Our data suggested crosstalk exist between c-Rel and the p53 pathway, including *MUT-TP53* induction at the transcriptional level in c-Rel^+^ ABC-DLBCL, the gain or loss of correlation with expression levels of genes involved in cell cycle (*p21, AURKB*), apoptosis (*BIRC5, BCL2L11*), TNF pathways (*TRAF2, TANK*), and tumor suppressor *TP63*. Concurrent c-Rel positivity and *TP53* mutation correlated with significantly worse patient survival. This may excel result from formation of different c-Rel/NF-κB dimers, functional alterations, posttranslational modification [59], or increased *REL* mutations in patients with MUT-p53 [60]. In contrast, WT-p53 and p63 may excel tumor suppressor function towards c-Rel signaling by cell cycle arrest, proapoptosis and BCR signaling inhibition therefore abolished the prognostic effect of c-Rel activation.

In summary, c-Rel nuclear expression but not *REL* amplification has an adverse prognostic effect in DLBCL which synergized with *TP53* mutations. c-Rel has distinctive and overlapping functions compared with other NF-κB subunits, and c-Rel/p65 and c-Rel/p50 dimers may be relevant for the oncogenic role of c-Rel in DLBCL. The biology revealed by c-Rel GEP signatures from this study has gained insight into the NF-κB pathways providing important information for further functional study, and suggest that therapeutic approaches targeting BCR, cell cycle, cytokine, and the p53 pathway, as well as BET inhibitors, but not proteasome inhibitors, may have clinical benefits in c-Rel^+^ DLBCL patients.

## PATIENTS AND METHODS

### Patients

This study included 460 patients with *de novo* DLBCL treated with standard R-CHOP immunochemotherapy consisting of rituximab plus cyclophosphamide, hydroxydaunomycin (doxorubicin), oncovin (vincristine), and prednisone. The diagnosis, review process, and cell-of-origin classification according to GEP or the immunohistochemical algorithms of Visco-Young and/or Choi have been described previously [36, 61]. Patients were excluded if they had HIV infection, primary cutaneous or nervous system DLBCL, primary mediastinal large B-cell lymphoma, or a history of low-grade B-cell lymphoma with transformation to DLBCL. This study was conducted in accordance with the Declaration of Helsinki, and informed consent was obtained from all patients whose tumor samples were used. The study protocol and material transfer agreement were approved by the institutional review boards of all participating centers. The overall study was approved by the Institutional Review Board of The University of Texas MD Anderson Cancer Center.

### Tissue microarray (TMA) and immunohistochemical assay

Immunohistochemical analysis for c-Rel, p50, p52, p65, RelB, p53, p63, Myc, Bcl-2, pSTAT3, pAKT, MDM2, and Ki-67 was performed on the TMA prepared with formalin-fixed, paraffin-embedded (FFPE) tissue blocks from all of the 460 DLBCL patients using methods previously described [36, 61, 67]. The results were analyzed independently by a group of hematopathologists (LL, CYO, AT, KHY), and disagreements were resolved by joint review with use of a multi-head microscope [70, 71].

### *TP53* mutation and fluorescence *in situ* hybridization (FISH) analysis

Genomic DNAs extracted from FFPE tissues were used for *TP53* exon sequencing analysis with use of the AmpliChip (Roche Molecular Systems) [36]. FISH analysis for *REL* amplification used a customer developed dual-color mix (Agilent Technologies, G100258R-8) consisting of a 2p16.1 (*REL*-locus) probe labeled with Spectrum Orange and a reference probe (chromosome 2 centromere) labeled with Spectrum Green. Dual-color FISH was performed on 4 micron sections of the TMAs. Fluorescence signals were scored by counting the number of single-copy genes and reference probe signals in 200 well-defined nuclei. High-level amplification was defined as the presence of either 6 gene signals or tight clusters of at least five gene signals per cell. Low level gains were considered when the ratio between REL and CEP2 signals exceeded 2. Cases were considered polysomic for chromosome 2e if the number of tumor cell nuclei with three or more signals exceeded the mean +3 s.d. of polysomic nuclei in the reference cases (i.e. 15%). Five tonsils were used as references.

Probes and methods of FISH analysis for *MYC, BCL2*, and *BCL6* translocation have been described previously [62].

### Gene expression profiling

Total RNAs were extracted from FFPE tissues and used for GEP by Affymetrix GeneChips array as described previously [36, 61]. GEP was achieved in 453 DLBCL patients. The CEL files are deposited in the National Center for Biotechnology Information Gene Expression Omnibus repository (GSE#31312).^61^ The microarray data were quantified and normalized by the frozen robust multiarray analysis (RMA) algorithm. The DEGs were identified by using multiple *t*-tests. Pathway analysis for the DEGs was performed with use of the Ingenuity Pathway Analysis software program (http://www.qiagen.com/ingenuity).

### Cell culture and coimmunoprecipitation

Human-derived DLBCL cell lines (MS, DB, LR, LP) were established from tissue biopsy or effusion specimens from patients as described previously [63, 64]. The cells were cultured in RPMI medium (Gibco, Rockville, MD) containing 15% fetal calf serum (FCS; Hyclone, Logan, UT). Coimmunoprecipitation was carried out as described previously [65, 66].

### Statistical analysis

The clinical and pathologic features at the time of presentation were compared between various DLBCL subgroups by using the Chi-square test. Correlation between expression of different genes or proteins was evaluated by the Spearman rank correlation method and unpaired *t* tests. Overall survival was calculated from the date of diagnosis to the date of last follow-up or death. Progression-free survival was defined as the time period from the date of diagnosis to the date of progression or death. OS and PFS curves of the various groups were analyzed by GraphPad Prism 6 (GraphPad Software, San Diego, CA) using the Kaplan-Meier method, and differences were compared with use of the log-rank (Mantel-Cox) test. Multivariate analysis was conducted by using the Cox proportional hazards regression model with the SPSS software (version 19.0; IBM Corporation, Armonk, NY). Any difference with a *P* value of < 0.05 was considered statistically significant.

## SUPPLEMENTARY FIGURES AND TABLES


