# Supplementary material for: Prognostic impact of c-Rel nuclear expression and REL amplification and crosstalk between c-Rel and the p53 pathway in diffuse large B-cell lymphoma
Source: Oncotarget. 2015 Jun 30;6(27):23157–80. doi: 10.18632/oncotarget.4319 (PMC4695110; doi:10.18632/oncotarget.4319)
Supplement: Supplementary file 1 [file oncotarget-06-23157-s001.pdf]

SUPPLEMENTARY FIGURES AND TABLES

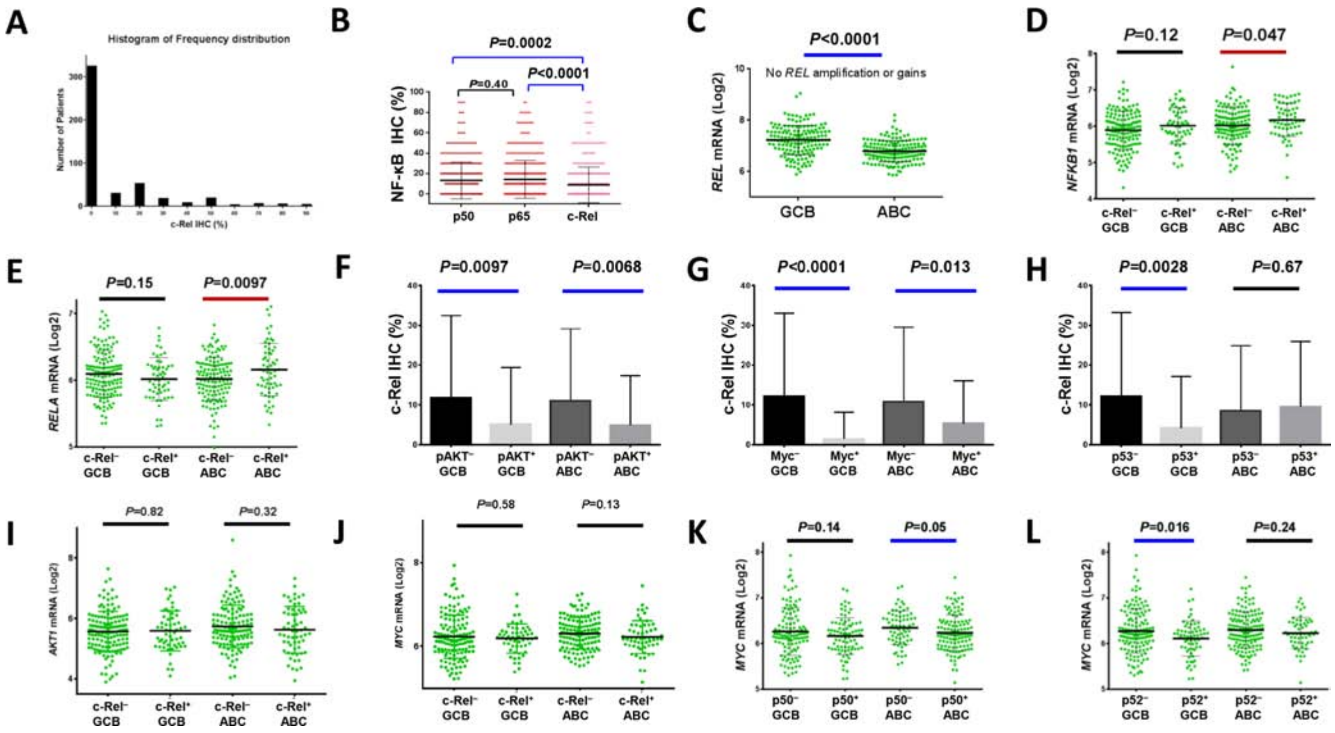

Supplementary Figure S1. A. Histogram of c-Rel nuclear expression in the DLBCL cohort. **B.** Expression level of c-Rel compared with p65 and p50 in DLBCL. **C.** GCB-DLBCL has significantly higher *REL* mRNA levels than ABC-DLBCL. **D-E.** In ABC-DLBCL, c-Rel positivity correlated with significantly higher *NFKB1* and *RELA* mRNA levels. **F-H.** pAKT, Myc and p53 overexpression correlated with significantly lower c-Rel levels in DLBCL (except in $p53^+$ ABC-DLBCL). **I-J.** c-Rel positivity did not correlate with *AKT1* and *MYC* mRNA levels. **K.** In ABC-DLBCL, $p50^+$ correlated with significantly lower *MYC* mRNA levels. **L.** In GCB-DLBCL, $p52^+$ correlated with significantly lower *MYC* mRNA levels. Note: red lines indicate significant upregulation whereas blue lines indicated downregulation.

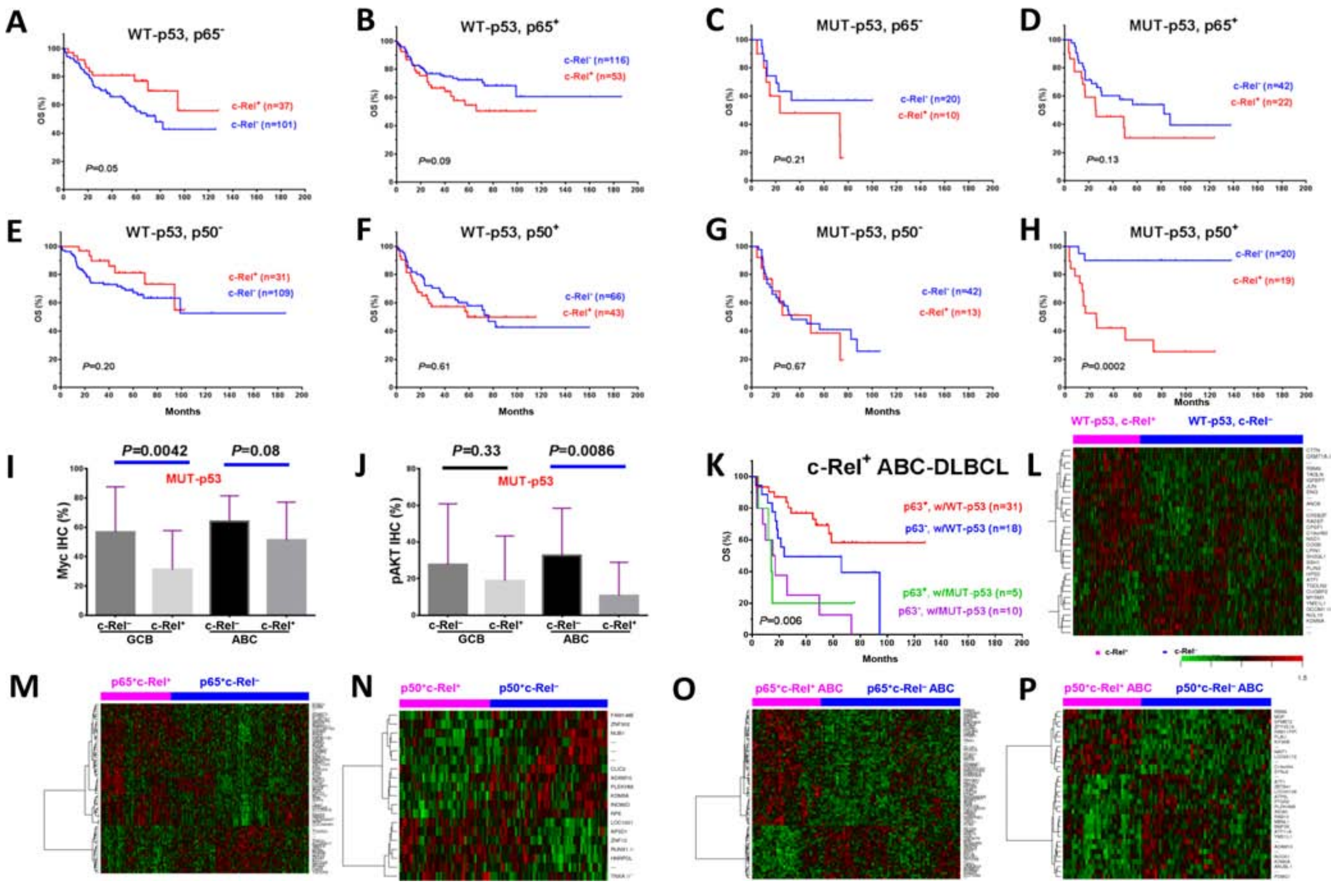

Supplementary Figure S2. A-D. In $p65^-$ DLBCL with wild-type-p53 (WT-p53), c-Rel nuclear expression correlated with significantly better survival. In contrast, in $p65^-$ DLBCL with mutated p53 (MUT-p53), c-Rel nuclear expression showed trend toward poorer survival. In $p65^+$ DLBCL, c-Rel nuclear expression appeared to correlate with poorer survival regardless of p53 mutation status. **E-H.** Impact of c-Rel nuclear expression in $p50^-$ or $p50^+$ DLBCL with a wild-type or mutated p53. In $p50^+$ DLBCL with MUT-p53, c-Rel nuclear expression correlated with significantly poorer survival. **I-J.** c-Rel positivity correlated with lower levels of pAKT and Myc in DLBCL with a mutated p53, most significantly in GCB- and ABC-DLBCL respectively. **K.** The correlation of p63 expression with better survival in patients with wild-type p53 was abrogated by p53 mutations in ABC-DLBCL. Note: blue lines indicated downregulation with significant or border-line *P* values. **L.** Heatmap by gene expression profiling analysis in DLBCL with WT-p53. **M-N.** Heatmaps by gene expression profiling analysis in DLBCL with concurrent c-Rel/p65 or c-Rel/p50 activation. **O–P.** Heatmaps by gene expression profiling analysis in ABC-DLBCL with concurrent c-Rel/p65 or c-Rel/p50 activation.

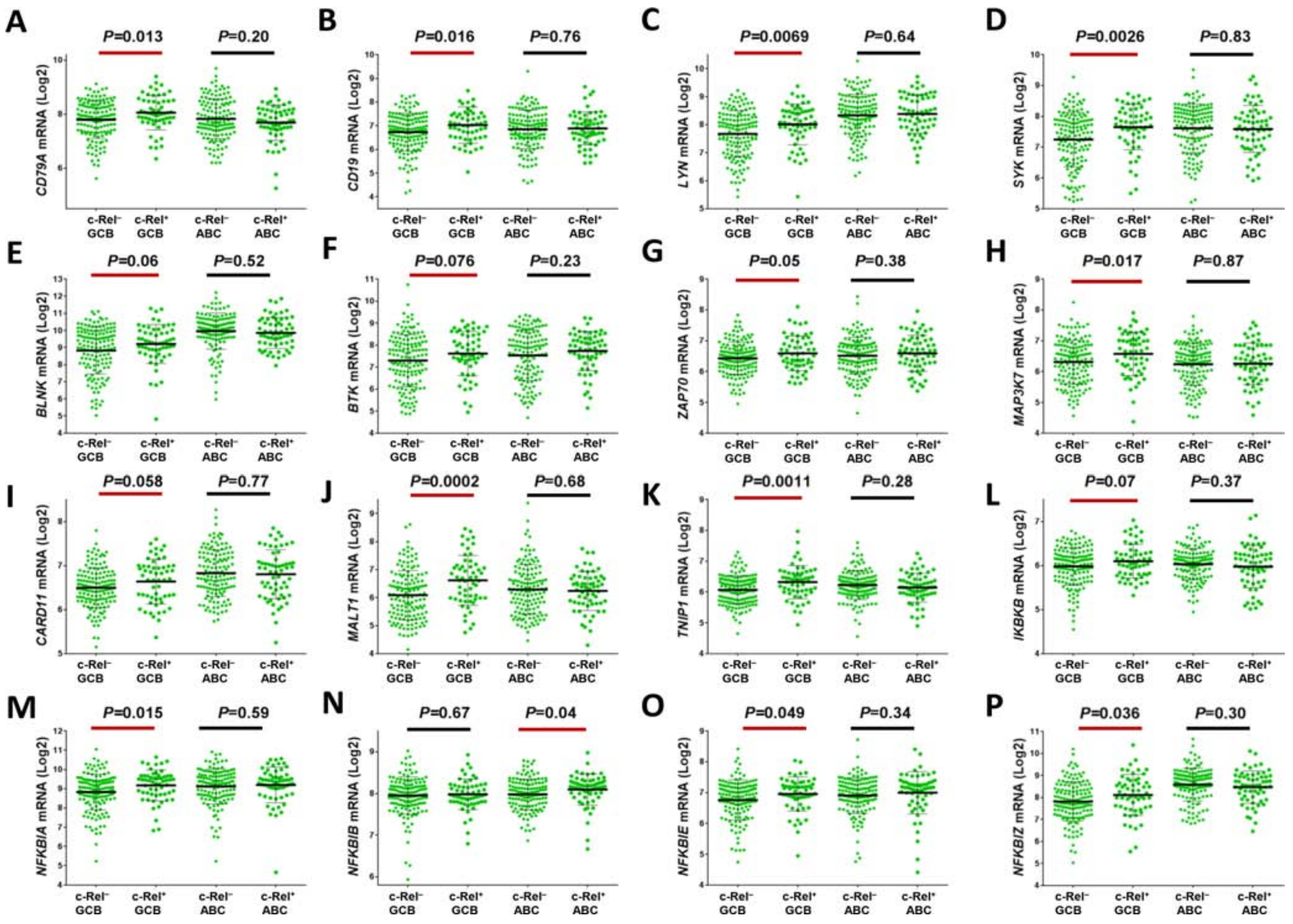

Supplementary Figure S3. Gene expression analysis of upstream regulators for c-Rel activation. **A-J.** Genes involved in the BCR signaling cascade, including *CD79A, CD19, LYN, SYK, CARD11, MALT1, BLNK, BTK, ZAP70*, and *MAP3K7*/*TAK1* were significantly upregulated in c-Rel^{+} compared with c-Rel^{-} GCB-DLBCL. **K.** *TNIP1* negatively regulates BCR, TNF, and NF-κB signaling was also significantly upregulated in c-Rel^{+} GCB-DLBCL. **L.** *IKBKB* encoding IKK*2* (but not *IKK1* gene) was upregulated in c-Rel^{+} GCB-DLBCL with a border line *P* value. **M.** *NFKBIA* (encoding IκBα) was significantly upregulated in c-Rel^{+} GCB-DLBCL. **N.** *NFKBIB* (encoding IκBβ) was significantly upregulated in ABC-DLBCL. **O.** *NFKBIE* (encoding IκBε) was significantly upregulated in c-Rel^{+} GCB-DLBCL. **P.** c-Rel positivity correlated with significantly higher *NFKBIZ* (encoding IκB-zeta) mRNA levels in GCB-DLBCL. Note: red lines indicate upregulation whereas blue lien indicated downregulation with significant or border-line *P* values.

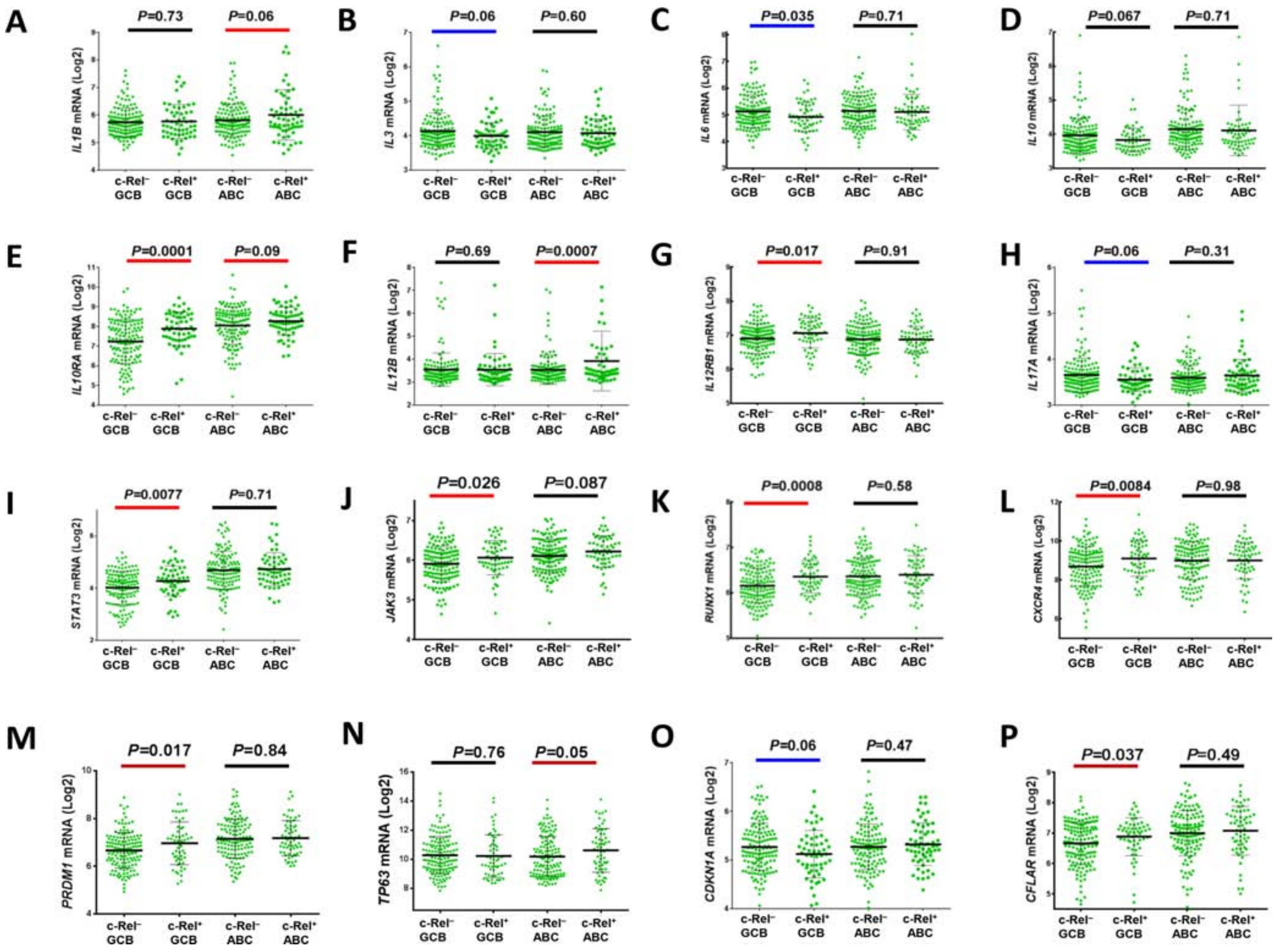

Supplementary Figure S4. Gene expression analysis correlating with c-Rel nuclear expression in GCB- and ABC-DLBCL. A-L. Differential expression of cytokine/chemokine related genes between c-Rel^{-} and c-Rel^{+} in GCB- or ABC-DLBCL. **M-O.** Differential expression of *PRDM1*/*BLIMP1, TP63* and *CDKN1A/p21* between c-Rel^{-} and c-Rel^{+} in GCB- or ABC-DLBCL. **P.** *CFLAR* was significantly upregulated in c-Rel^{+} GCB-DLBCL. Note: red lines indicate upregulation whereas blue lines indicated downregulation with significant or border-line *P* values.

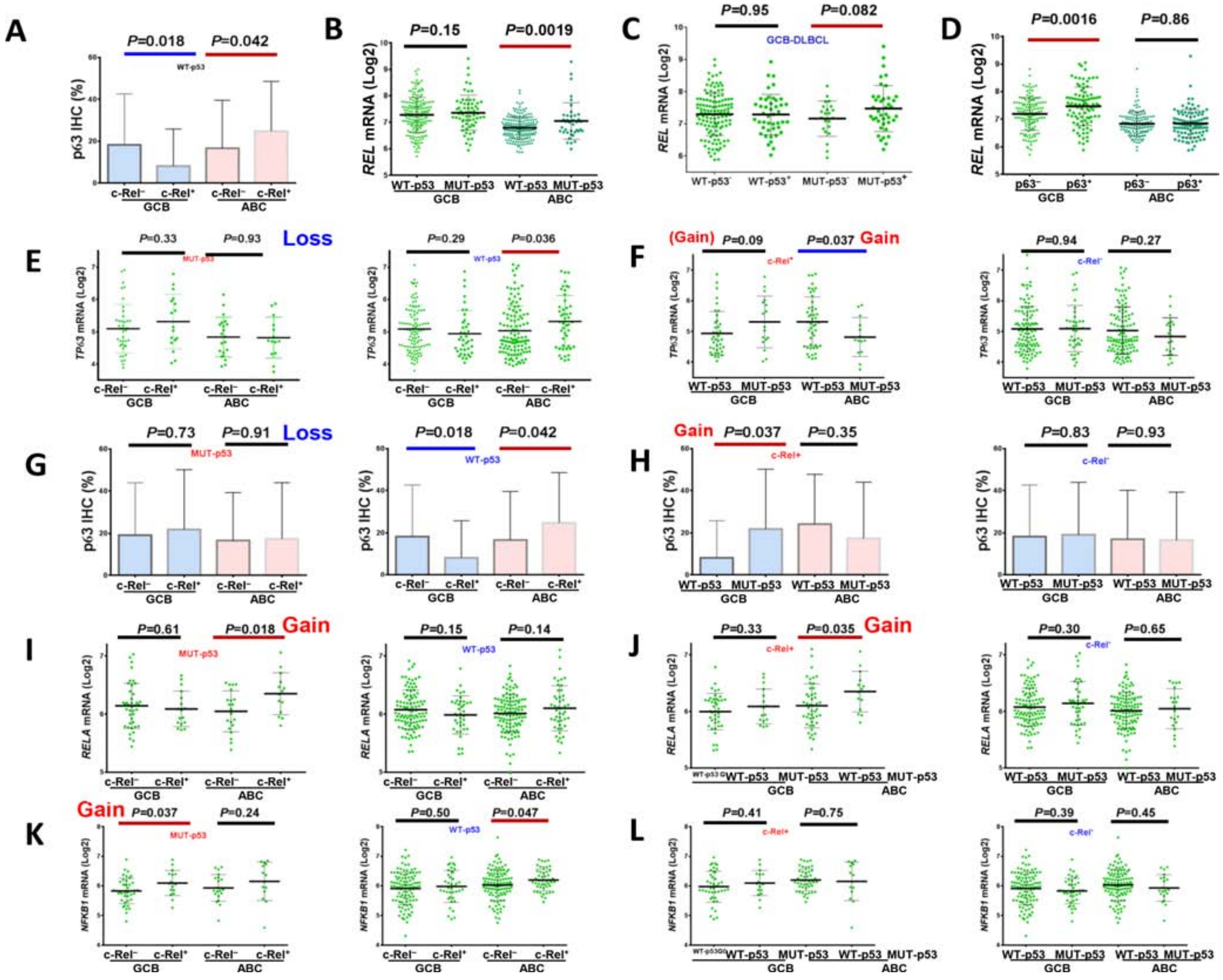

Supplementary Figure S5. A. In ABC-DLBCL with WT-p53, c-Rel nuclear expression was associated with significantly higher p63 protein levels. **B.** In ABC-DLBCL, *TP53* mutations were associated significantly higher *REL* mRNA levels. **C.** In GCB-DLBCL, expression of MUT-p53 was associated with higher *REL* mRNA levels with a marginal *P* value. **D.** In GCB-DLBCL, p63 expression correlated with significantly higher *REL* mRNA. **E-H.** c-Rel appeared to lose function in upregulating *TP63* in ABC-DLBCL when concurrent with MUT-p53, suggested by analysis at the transcription and protein levels. **I.** c-Rel nuclear expression significantly correlated with *RELA* upregulation in ABC-DLBCL with MUT-p53, but not in ABC-DLBCL with WT-p53. **J.** *TP53* mutations significantly correlated with *RELA* upregulation in ABC-DLBCL with c-Rel nuclear expression, but not in ABC-DLBCL without c-Rel nuclear expression. **K-L.** c-Rel nuclear expression significantly correlated with *NFKB1* upregulation in GCB-DLBCL with MUT-p53, and in ABC-DLBCL with WT-p53. The expression pattern of *NFKB1* in p53 mutants did not show significant changes with or without c-Rel nuclear expression.

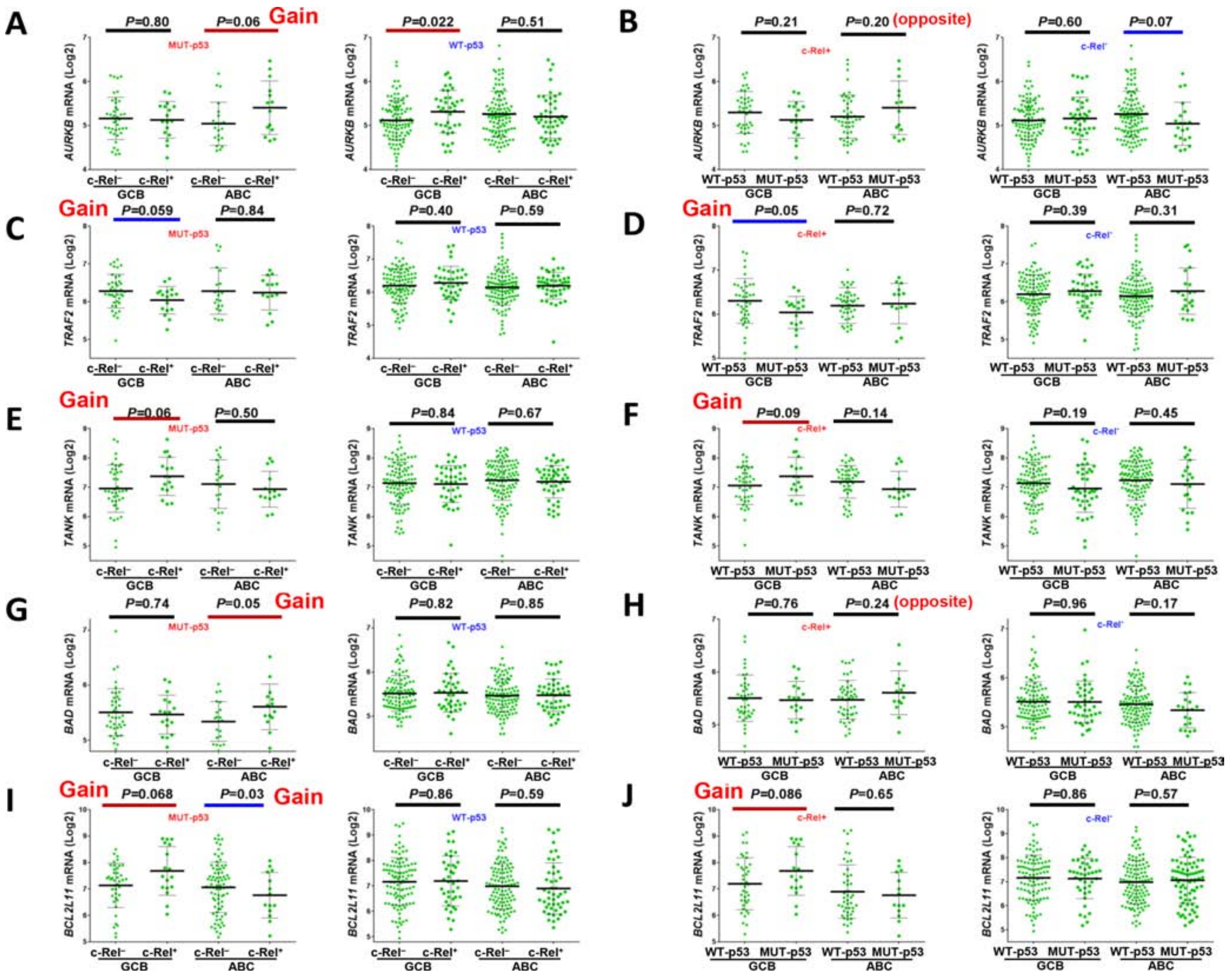

Supplementary Figure S65. A. In ABC-DLBCL with c-Rel nuclear expression, c-Rel nuclear expression was associated with higher *AURKB* transcription (marginal *P* value); in contrast in ABC-DLBCL without c-Rel nuclear expression, c-Rel nuclear expression was associated with higher *AURKB* transcription in GCB-DLBCL and not in ABC-DLBCL. **B.** In ABC-DLBCL with c-Rel nuclear expression, *TP53* mutations appeared to be associated with higher *AURKB* transcription (non-significant *P* value in this small cohort); in contrast in ABC-DLBCL without c-Rel nuclear expression, *TP53* mutations were associated with lower *AURKB* transcription (border-line *P* value). **C.** c-Rel nuclear expression correlated with *TRAF2* downregulation in GCB-DLBCL with MUT-p53, but not in GCB-DLBCL with WT-p53. **D.** *TP53* mutations correlated with *TRAF2* downregulation in GCB-DLBCL with c-Rel nuclear expression, but not in GCB-DLBCL without c-Rel nuclear expression. **E.** c-Rel nuclear expression correlated with *TANK* upregulation in GCB-DLBCL with MUT-p53, but not in GCB-DLBCL with WT-p53. **F.** In GCB -DLBCL with c-Rel nuclear expression, *TP53* mutations appeared to be associated with higher *TANK* transcription (marginal *P* value); in contrast in GCB-DLBCL without c-Rel nuclear expression, p53 mutant group appeared to have lower *TANK* transcription. **G.** c-Rel nuclear expression correlated with *BAD* upregulation in ABC-DLBCL with MUT-p53, but not in ABC-DLBCL with WT-p53. **H.** In ABC-DLBCL with c-Rel nuclear expression, *TP53* mutations appeared to have higher *BAD* transcription; in contrast in ABC-DLBCL without c-Rel nuclear expression, *TP53* mutations appear to have lower *BAD* transcription. **I.** c-Rel nuclear expression correlated with *BCL2L11* upregulation in GCB-DLBCL with MUT-p53 (but not in WT-p53 group), and *BCL2L11* downregulation in ABC-DLBCL with MUT-p53 (but not in WT-p53 group). **J.** *TP53* mutations appeared to be associated with higher *BCL2L11* expression in GCB-DLBCL with c-Rel nuclear expression, but not in GCB-DLBCL without c-Rel nuclear expression. Note: red lines indicate upregulation whereas blue lines indicated downregulation with significant or border-line *P* values.

Supplementary Table S1. Gene signatures of c-Rel$^+$ in patients with GCB-DLBCL (false discovery rate threshold: 0.30)

c-Rel$^+$ *vs.* c-Rel$^-$ GCB-DLBCL

Function	Upregulated	Downregulated
**Mitogen, cytokine, growth factor, receptors, signaling transduction**	*CAB39, DUSP10, DKK3*	
**Gene expression, transcription**	*JUN, FOXP1, FOXO3, BRD2*	*TCERG1, ZNF267, GTF2H2, ZNF614, POLR1B*
**Posttranscriptional regulation, transportation, degradation**	*RBM5, P4HB, UBA7, ST13, AP3D1, SNX19*	
**Actin, cytoskeleton, cell morphology, adhesion, extracellular matrix, migration**	*S100A4, TPM4, ACTG1, RHOA, SSH2, SSH1, WASF2, CAST, MYO9B, EML3*	
**Metabolism, redox**	*NADSYN1, SERINC1, POMT2*	
**Differentiation**	*PRDM1*	*EPO*
**Autoimmune**	*KIAA1109*	
**Unknown function**	*LOC100294335 / LOC644397 / LRRC37A*	*C1orf49, LOC100131088, NOL10*

Supplementary Table S2. Gene signatures of c-Rel$^+$ in the patients with p50$^+$ DLBCL (false discovery rate threshold: 0.30), and in the patients with p65$^+$ DLBCL (false discovery rate threshold: 0.10)

	p50$^+$c-Rel$^+$ *vs.* p50$^+$c-Rel$^-$		p65$^+$c-Rel$^+$ *vs.* p65$^+$c-Rel$^-$	
**Function**	**Upregulated**	**Downregulated**	**Upregulated**	**Downregulated**
**Mitogen, cytokine, growth factor, receptors, signal transduction, NF-κB activation**		*CLIC2, ADAM10*	*CTGF, AEBP1, EFEMP1, GPR124, IGFBP5, RASEF, KLF6, ANXA2P2, TBC1D20, FARP1, ENG*	
**DNA repair**				*RECQL*
**Gene expression, transcription and translation regulation**	*ZNF12, RUNX1, HNRPDL*	*ZNF302, INO80D, KDM5A*	*JUN, BRD2, SRRM2, ANKRD11, MXD4, SFMBT2, SBNO2, MLLT10*	*CHD2, ATF1, MYNN, ZBTB2, TCERG1*
**Actin, cytoskeleton, cell morphology, adhesion, extracellular matrix, migration**	*SH3D19*	*FAM148B*	*CALD1, DNM2, SSH1, BGN, PAFAH1B1, BTBD7*	
**Protein sorting, protein and vesicle's trafficking, transportation, chaperone**	*AP3D1*		*GGA3, NRBP1, VPS53, AP3D1*	*SEC23B, TNPO1, PLDN, TGOLN2, SLC25A17*
**Metabolism, redox**		*RPE*	*NADSYN1, COG1, EPHX1, SLC25A16, SIRT3*	*PPARA*
**Tumor suppressor, necrosis**			*MEG3, ALKBH7*	
**Degradation**		*NUB1*	*UBA7*	
**Immune response**			*SPN*	
**Unknown function**	*NPIPL3, TNXA/TNXB*	*PLEKHM3*	*BSG, LOC339047, NPIP, LOC399491, FKSG49*	*JRKL, NARG2, NOL10*

Supplementary Table S3. Gene signatures of c-Rel$^+$ in patients with p50$^+$ ABC-DLBCL (false discovery rate threshold: 0.30), and in the patients with p65$^+$ ABC-DLBCL (false discovery rate threshold: 0.10)

	p50$^+$ c-Rel$^+$ ABC *vs.* p50$^+$ c-Rel$^-$ ABC		p65$^+$ c-Rel$^+$ ABC *vs.* p65$^+$ c-Rel$^-$ ABC	
**Function**	**Upregulated**	**Downregulated**	**Upregulated**	**Downregulated**
**Mitogen, cytokine, growth factor, receptors, signal transduction, NF-kappaB activation**		*BMP2K*	*AEBP1, IGFBP5, GPR124, SIGIRR, SERPINE1, SEMA4C, MRVI1, ENG, C7orf59, DIP2A*	*SETD6, GNB5*
**DNA replication, cell cycle**		*INO80*	*PURB*	*MAK*
**Gene expression, transcription and translation regulation**	*RBM9, SFMBT2*	*ATF1, MBNL1, ZBTB41, KDM5A*	*HIST2H2AA3 / HIST2H2AA4, TFE3, C16orf42, RRBP1, RBM9, EYA2, SBNO2, ZNF286A, LHX6, LMNA, MLLT10, HOXC8*	*ATF1, EIF1B*
**Actin, cytoskeleton, cell morphology, adhesion, extracellular matrix, migration**	*MGP, KIF26B, PLAU*	*ADAM10, ROCK1*	*TAGLN, SERPINH1, LDB3, KIF26B, LTBP2, BGN, BTBD7, PLAU, HSPB1, VWA1, PPFIBP1*	*ADAM10, ROCK1, PTPRM*
**Protein sorting, transportation, vesicle's trafficking, chaperone**	*RAB11FIP3, ZFYVE19*	*RAB10, PSMG1*	*RAB11FIP3, KIAA0415*	*SLC12A9, HERC4, SERP1, SEC23B, DNAJB14, PSMG1*
**Metabolism, redox**		*ATP11A, YME1L1, ATP5L, PTGR2*	*GLYR1*	*DCK, PDE12, TMX3*
**Tumor suppressor, apoptosis**	*NAIF1*		*MEG3*	*TNFAIP8*
**Unknown function**	*C19orf44, LOC651721, SYNJ2*	*LOC401397, PLEKHM3, ANUBL1*	*LOC728264, ODF3B, LOC651721, BTNL8, KIAA0894*	*PLEKHM3, WDR89*

Supplementary Table S4. Gene signatures of c-Rel$^+$ DLBCL in patients with WT-p53 (false discovery rate threshold: 0.15)

c-Rel$^+$ (*vs.* c-Rel$^-$) DLBCL with WT-p53		
**Function**	**Upregulated**	**Downregulated**
**Signal transduction, receptors**	*IGFBP7, ENG, ANO8*	
**Cell cycle**	*SH3GL1*	
**Gene expression, transcription and translation regulation**	*JUN, CPSF1, RBM9, CREBZF, NSD1*	*CUGBP2, ATF1, KDM5A, GCOM1/ GRINL1A, MYSM1*
**Actin, cytoskeleton, adhesion, migration**	*TAGLN, SSH1, CTTN*	
**Protein processing, transportation, vesicle's trafficking**	*PLIN3, RASEF, COG5*	*HPS3, TGOLN2*
**Metabolism**	*LPIN1, CKMT1A/B*	*YME1L1*
**Unknown function**	*C19orf60*	*NOL10*
